# Inhibition of the CXCL9-CXCR3 axis suppresses the progression of experimental apical periodontitis by blocking macrophage migration and activation

**DOI:** 10.1038/s41598-021-82167-7

**Published:** 2021-01-28

**Authors:** Tatsuya Hasegawa, V. Venkata Suresh, Yoshio Yahata, Masato Nakano, Shigeto Suzuki, Shigeki Suzuki, Satoru Yamada, Hideki Kitaura, Itaru Mizoguchi, Yuichiro Noiri, Keisuke Handa, Masahiro Saito

**Affiliations:** 1 Division of Operative Dentistry, Department of Ecological Dentistry, Graduate School of Dentistry, Tohoku University, 4-1 Seiryo-machi, Aoba-ku, Sendai, Miyagi 980-8575 Japan; 2 Division of Periodontology and Endodontology, Department of Ecological Dentistry, Graduate School of Dentistry, Tohoku University, Sendai, Miyagi Japan; 3 Division of Orthodontics and Dentofacial Orthopedics, Graduate School of Dentistry, Department of Community Social Dentistry, Tohoku University, Sendai, Miyagi Japan; 4 Division of Cariology, Operative Dentistry and Endodontics, Department of Oral Health Science, Graduate School of Medical and Dental Sciences, Niigata University, Niigata, Japan; 5 Division of Oral Biochemistry, Department of Oral Science, Graduate School of Dentistry, Kanagawa Dental University, Yokosuka, Kanagawa Japan

**Keywords:** Immunology, Molecular biology

## Abstract

Apical periodontitis (AP) is an acute or chronic inflammatory disease caused by complex interactions between infected root canal and host immune system. It results in the induction of inflammatory mediators such as chemokines and cytokines leading to periapical tissue destruction. To understand the molecular pathogenesis of AP, we have investigated inflammatory-related genes that regulate AP development. We found here that macrophage-derived CXCL9, which acts through CXCR3, is recruited by progressed AP. The inhibition of CXCL9 by a CXCR3 antagonist reduced the lesion size in a mouse AP model with decreasing IL-1β, IL-6 and TNFα expression. The treatment of peritoneal macrophages with CXCL9 and LPS induced the transmigration and upregulation of osteoclastogenic cytokines such as IL-1β, IL-6 and matrix metalloprotease 2, a marker of activated macrophages. This suggests that the CXCL9-CXCR3 axis plays a crucial role in the development of AP, mediated by the migration and activation of macrophages for periapical tissue destruction. Our data thus show that CXCL9 regulates the functions of macrophages which contribute to AP pathogenesis, and that blocking CXCL9 suppresses AP progression. Knowledge of the principal factors involved in the progression of AP, and the identification of related inflammatory markers, may help to establish new therapeutic strategies.

## Introduction

Apical periodontitis (AP) is an inflammatory disease accompanied by bone destruction in the periapical tissues of the jaw and is caused by bacterial infection from the dental pulp^1^. The onset of AP is initiated by pathogenic bacteria and their components or foreign substances released from the infected root canal^2^. These factors stimulate periodontal ligament cells to express inflammatory related molecules such as cytokines, chemokines, and thereby promote the expansion of capillary vessels to infiltrate inflammatory cells, including leukocytes, macrophages and lymphocytes^3^. Subsequently this pathological environment recruits’ osteoclasts to the apex of the tooth root that cause bone destruction, and alters the progression of the disease^4,5^. Histologically AP is characterized by the formation of granulation tissue in response to acute inflammation, and may eventually develop into a radicular cyst^6^. Although reducing the pathogen load from the root canal typically prevents reinfection and leads to healing of the AP, the complex root canal system and host immune system sometimes interfere with these processes resulting in therapy-resistant AP lesions that induce continuous apical bone tissue destruction^7,8^. In addition, the cumulative evidence to date has reinforced the notion that there is a positive correlation between the pathogenesis of endodontic infection and systemic diseases such as cardiovascular disease^9^, diabetes^10,11^, bowel disease^12^. This is due to overactivation of the host immune system resulting in alterations or influences upon systemic diseases, enhanced inflammation of the periapical area, and bone destruction^13^. Thus, a fuller understanding of the pathogenesis of AP, especially of persistent cases and instances in which other systemic diseases are present, may identify potential new therapeutic targets.

Numerous animal model studies and clinical investigations on the pathogenesis of AP have now shown the involvement of inflammatory cytokines and chemokines in this disorder but the details of the underlying mechanisms still remain to be determined^14,15^. These cellular factors are initiated in AP as a result of innate immunity which can recognize the invading microorganisms and initiate host responses including the recruitment of leukocytes, monocyte derived macrophages, natural killer cells and other cells from the blood to eliminate the microbes from the infected root canal^15,16^. Among these responsive cells, macrophages can phagocytose pathogens, function as antigen-presenting cells (APCs) and secrete different types of cytokines, including interleukin-1 (IL-1), IL-6, and tumor necrosis factor α (TNFα), and have a role in the progression of AP^17,18^. During the initial stages of the innate immune response, tissue-resident macrophages act as a first line of defense against pathogens and secrete inflammatory cytokines and chemokines. Macrophage markers such as CD11b-, F4/80-, and CD64-positive cells were found in the periapical area, indicating their involvement in the onset of AP in the mouse^19,20^. At later stages of the immune response, the macrophages that differentiate from circulating monocytes migrate and play a role in the protection against pathogens^19^. These macrophages have dual functions in the regulation of inflammation at the lesion site, depending on their activation, which are as classically (M1) or alternatively (M2) polarized macrophages that exhibit pro- and anti-inflammatory functions, respectively. The M1 macrophages produce pro-inflammatory cytokines such as interleukin-1β (IL-1β), IL-6, IL-12, IL-23, TNFα, whereas M2 macrophages produce anti-inflammatory cytokines such as IL-10 and TGFβ^19,21^. Various previous studies have demonstrated that macrophages are abundant in AP lesions, and it is theorized that the migration and activation of macrophages play a crucial role in the development of AP^22,23^. Recent reports have reported that the switching of macrophages from an inflammatory phenotype towards their differentiation into osteoclasts occurs in the presence of pro-inflammatory cytokines that are well known to affect the differentiation of osteoclasts for bone destruction^24^. During the onset of AP, the local production of the osteoclastogenic factor RANKL as well as pro-inflammatory cytokines were increased, indicating that pathogenic bone resorption was required for development of the lesion^25^. Thus, deciphering the process of macrophage activation and recruitment may provide insights into the pathogenesis of AP.

Recently, much interest has been focused on chemokines, a family of small molecular weight proteins that act as potent mediators of inflammation and are considered to be critical factors involved in the migration and activation of macrophages during various inflammatory diseases including AP^14,26,27^. Chemokine ligands are divided into four groups (XC, CC, CXC and CX3C) and chemokine receptors comprise a group of more than 20 rhodopsin-like seven transmembrane-spanning receptors including CC receptors, CXC receptors, and typical chemokine receptors that show non G-protein-coupled signaling^28^. Chemokines and their receptors enable the selective tissue-specific recruitment of circulating monocyte subsets to the area of inflammation^29,30^. Chemokines are also considered to be important signaling molecules for macrophage migration and activation under pathological conditions^29–32^. Macrophages are involved in the induction of chemokines, in which M1 macrophages express Th1 cell-attracting chemokines such as CXCL9 and CXCL10 as a result of activation by pro-inflammatory cytokines such as interferon gamma (IFNγ) and TNFα^33,34^. Furthermore, it has been reported that CXCL9 and CXCL10 act as macrophage chemoattractants in acute and chronic cardiac inflammation^35^ and arthritis^36,37^, implicating these chemokines in inflammatory disease. Additionally, in the case of AP development, the upregulation of CXCL9 and CXCL10 has been detected in a mouse model and human samples^38,39^. Similarly, osteoclast precursors express CXCR3 that can interact with the corresponding chemokine ligand CXCL9 and thereby promote osteoclast migration and differentiation^40^.

In our present study, we have characterized the expression of chemokines in the periapical area during AP development to elucidate their functional role in maintaining and establishing these lesions. We have found that the chemokine CXCL9 is upregulated during the development of AP, and that antagonizing CXCR3 suppresses the bone destruction that typifies this disease by inhibiting various pro-inflammatory cytokines that are also involved in destruction of alveolar bone.

## Material and methods

### Animal experiments

All animal experimental procedures conformed to "Regulations for Animal Experiments and Related Activities at Tohoku University", were reviewed by the Institutional Laboratory Animal Care and Use Committee of Tohoku University, and were finally approved by the President of the University (Permit No. 2019 SHIDO-037). Animal experiments carried out in compliance with the ARRIVE guidelines. Wild-type (WT) C57BL/6N mice were purchased from Japan SLC, Inc (Shizuoka, Japan).

### Apical periodontitis mouse model

A mouse model of periapical lesions was generated in accordance with the previously published protocol^41–43^. Briefly, the left mandibular first molar pulp in 10-week-old male mice was exposed using a dental hand piece (VIVAMATE G5, NSK, Tochigi, Japan) with a #1/4 round bur (ISO 005; Dentsply Maillefer, Ballaigues, Switzerland) under a surgical microscope (OPMI 19FC, Carl Zeiss, Oberkochen, Germany). The dental pulp was removed with a #06K-file (Dentsply Maillefer) and root canals were left open to the oral environment to induce periapical lesions. The mandibles of the mice were dissected at 1, 3, 7, 14 and 28 days after pulp exposure, and fixed in 4% paraformaldehyde in PBS overnight at 4 °C. We then conducted µCT scanning and 3D construction to measure the lesion volumes, after which the mandibles were subjected to histological analysis.

### Histological analysis

Harvested tissues were fixed with 4% paraformaldehyde and decalcified using a solution consisting of 20% formic acid and 10% citric acid for 3 days, or 10% EDTA in PBS for 10 days, to be processed for HE staining and immunohistochemical analysis or TRAP staining, respectively. Subsequently, 5 µm sections were stained with hematoxylin and eosin and observed under a Leica DM6000B and Leica MC 170HD microscope (Leica, Wetzlar, Germany). For immunofluorescence, the samples were dehydrated in a 20% sucrose solution, embedded in OCT compound (Sakura Tissue-Tek, 4583), and sectioned at an 8 µm thickness using a cryostat (CM3050S, Leica). The sections underwent blocking with 2% bovine serum albumin (BSA) before being incubated with primary antibodies at 4 °C overnight and secondary antibodies at RT for 1 h. The following primary antibodies were used: rat anti-Ly6g antibody (Abcam; ab25377, 1:100), goat anti-MMP9 (R&D Systems Inc., Minneapolis, MN; AF909, 1:100), rat anti-Mac2 antibody (Abcam; ab53082, 1:300), goat anti-CXCL9/MIG antibody (R&D Systems; AF-492, 1:200), rabbit anti-Cathepsin K antibody (Abcam; ab19027, 1:100) donkey anti-rat IgG (Life Technologies, Carlsbad, CA) and donkey anti-goat IgG (Life Technologies). The following secondary antibodies were used: Alexa Fluor 594 donkey anti-rabbit IgG (Jackson ImmunoResearch; 711-585-152, 1:500) and Alexa Fluor 488 donkey anti-goat IgG (Jackson ImmunoResearch; 705-545-003, 1:300). The sections were counterstained with Hoechst33342 dye (Dojindo; H342, 1:1000) and mounted using Fluoromount (Life Technologies, K024). The samples were then observed under a confocal laser scanning microscope (LSM510; Carl Zeiss, Oberkochen, Germany). Immunohistochemical analysis of CD11b was performed as described previously using rabbit anti-CD11b (NB110-89474, 1:100) polyclonal antibody^44^. For the quantification of CD11b-positive cells, we selected 10 independent areas in the periapical lesion and quantified these regions using ImageJ 1.50i software (NIH, Bethesda, MD). Statistical analyses were performed as described below.

### TRAP staining

TRAP staining solution was prepared by adding the following solutions and reagents: 180 ml of 0.1 M sodium acetate (pH 5.0), 20 ml of 0.5 M sodium tartrate acid (pH5.0), 2 ml of N-N-dimethylformamide, 100 mg of Fast Red Violet LB Salt (Sigma-Aldrich, St Louis, MO), and 20 mg of Naphthol AS-MX Phosphate (Sigma-Aldrich). This staining solution was then transferred onto the sections that were maintained in a dark chamber at 37 °C for 40 min. After incubation, the sections were counterstained with hematoxylin for 30 s.

### Administration of CXCR3 antagonist in the mouse AP model

The AP mice received a gavage administration of SCH546738 (30mpk, diluted to 0.2 ml using 0.5% w/v methylcellulose) or a 0.5% w/v methylcellulose (vehicle) every 2 days from day 2 until the day before sacrifice. The mice without AP received a 0.5% w/v methylcellulose vehicle every 2 days from day 2 until the day before sacrifice and used as a control. The CXCR3 antagonist SCH546738 was purchased from MedChemExpress (Monmouth Junction, NJ).

### Effect of CXCL9 on activation of peritoneal macrophages

Peritoneal macrophages were prepared as described previously^45^. Briefly, cell suspensions were divided into four groups in a 6-well plate, cultured for 1 h, and then washed twice to remove non-adherent cells and used as peritoneal macrophages. These cells were later treated with LPS (100 ng/mL), recombinant mouse (rm) CXCL9 (100 ng/mL, 492-MM; R&D Systems) or LPS (100 ng/mL) and rmCXCL9 (100 ng/mL) in the presence of 10% FBS for 24 h. After incubation, the cells were assayed by the gene expression analysis of *Cxcl9, Cxcl10, Il1b, Il6, Mmp2, Tnf* (primer lists: sTable 1), as described previously^44^.

### Effect of CXCL9 on the chemotaxis of macrophages

To perform the migration assay, peritoneal macrophages were inoculated into a QCM Chemotaxis Assay 24-well plate (ECM506, EMD Millipore Corp, Billerica, MA), and then treated with rmCXCL9 (100 ng/mL), LPS (100 ng/mL) or rmCXCL9 and LPS in the presence of 10%FBS for 24 h. Quantification of the migrated cells was performed using a VersaMax Microplate Reader in accordance with the manufacturer's protocol (Molecular Devices, Sunnyvale, CA).

### Effect of CXCL9 on osteoclast differentiation

Osteoclast precursors were prepared as described previously^45,46^. These cells were then seeded at 5 × 10^4^ cells per 200 µL of medium in a 96-well plate and cultured in medium containing M-CSF alone (100 ng/mL), M-CSF (100 ng/mL) and rmRANKL (10 ng/mL; 315–11, PeproTech Rocky Hills, NJ), M-CSF (100 ng/mL) and rmCXCL9 (100 ng/mL), or rmCXCL9 (100 ng/mL), M-CSF (100 ng/mL), and rmRANKL (10 ng/mL). After 5 days, the cell cultures were fixed in a 10% formalin for 30 min and permeabilized with 0.2% Triton X-100 for 5 min at RT. TRAP-positive cells with three or more nuclei were considered to be osteoclasts and were counted under a light microscope. The quantification of TRAP-positive cells was done using ImageJ 1.50i software (NIH, Bethesda, MD). After incubation, the cells were assayed by the gene expression analysis of *Cxcr3*, *Ctsk*, *Calcr*, *Nfatc1*, *OC-Stamp* and *Mmp9* (primer lists: sTable 1), as described previously^44^.

### Statistical analysis

The study results were all tested in triplicate. The means and standard deviations were thereby calculated and the statistical significance of the differences between groups were examined using one-way analysis of variance (ANOVA), followed by a Dunnett’s multiple comparison test, or a multiple comparison test followed by a Tukey–Kramer test. Test results were considered significant at probability (p) values < 0.05.

## Results

### Development of a mouse AP model and analysis of the host-immune/inflammatory response

To analyze tooth root formation in C57BL6 mice, the root canal length and width of the mandibular first molar were investigated. For this purpose, µCT analysis of the mandibular first molar in the experimental mice was commenced from 4 weeks until 12 weeks (sFig. 1a). The average length (RL) of the mesial root canal was 1.1 mm at 4 weeks and increased to 1.4 mm at 9 weeks. The mean root canal width (RW) was 0.27 mm at 4 weeks but decreased to 0.10 mm at 9 weeks (sFig. 1b). These results suggested that the open apices of the mesial and distal roots were completely closed by 9 weeks, indicating the completion of root development. Hence, we decided to use 10-week-old mice for all further experiments.

**Figure 1:**
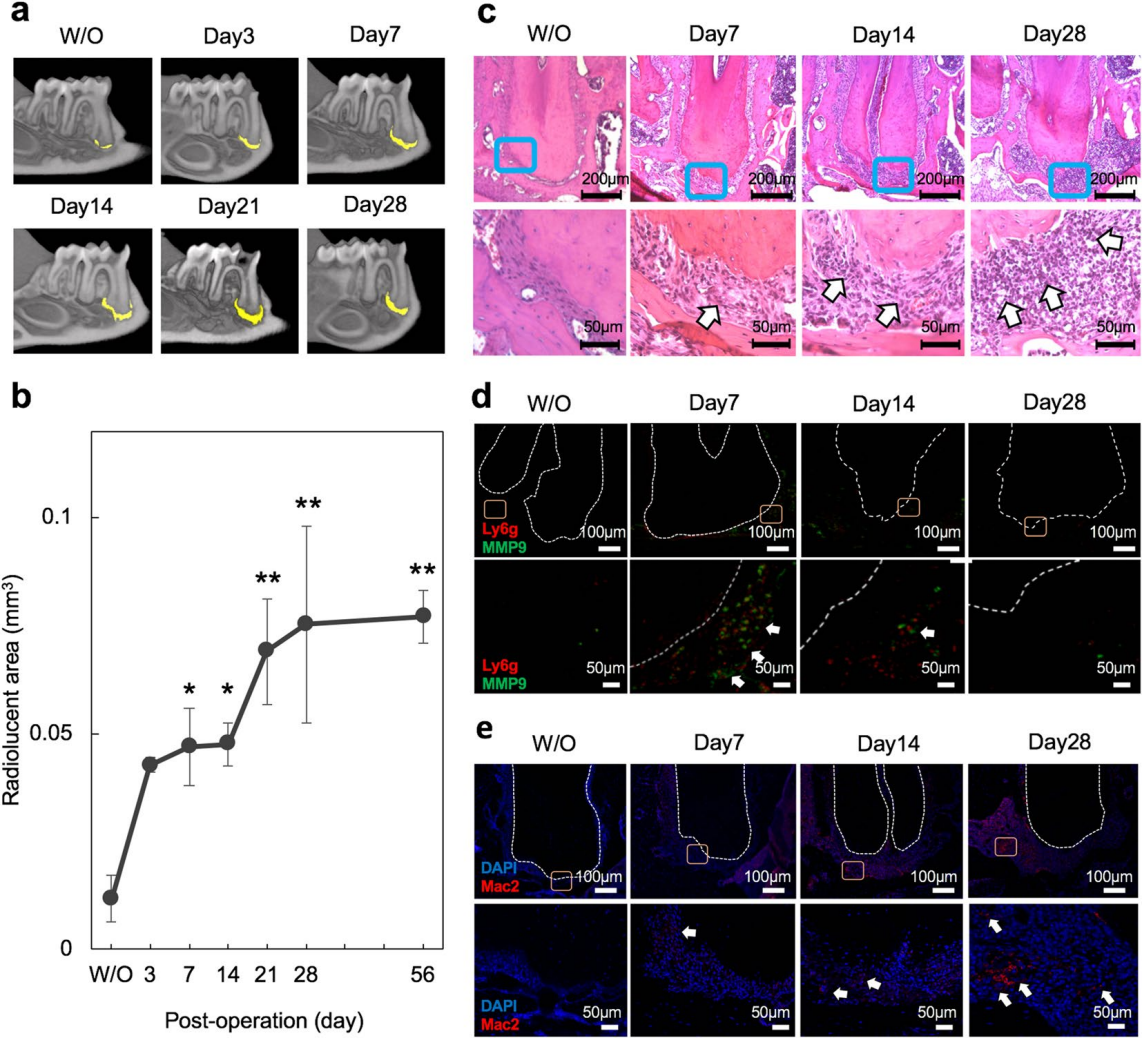
Establishment of a mouse AP model and analysis of lesion formation over time. (**a**) Representative μCT images of AP development in the mouse model until 28 days after pulp exposure. The radiolucent area is indicated by the yellow color and was determined using TRI/3D-BON software. (**b**) Quantitative analysis of the radiolucent area (mm^3^) from the μCT images. Statistical analysis was done using the Dunnett test to compare group with and without (W/O) exposure; *p < 0.05, **p < 0.01 (mean ± SD, n = 3 per group). (**c**) HE staining of AP development in the mouse model until 28 days after pulp exposure. The arrow denotes inflammatory cell filtration. (**d**) Representative images showing the immunostaining of Ly6g- and MMP9-positive neutrophils (arrows). (**e**) Representative images showing the immunostaining of Mac2-positive macrophages (arrows).

It is well documented that AP is caused by oral bacterial infection which stimulates host inflammatory/immune responses^1–5^. Sequential changes in inflammatory responses are required for bone destruction and we thus performed spatial temporal investigations during the onset of AP in our mouse model. We thus investigated lesion development and the inflammatory process in our mouse AP model (sFig. 2a), as described previously^42,43^. A 3D reconstruction image (axial, coronal and sagittal views) of the mesial root of the mandibular first molar revealed an apical radiolucent area (highlighted in yellow) on day 3 that gradually increased up to 28 days (Fig. 1a, sFig. 2b). The size of this area was 0.043 mm^3^ on day 3 and progressively increased to 0.048 mm^3^ and 0.075 mm^3^ at days 14 and 28 after exposure, respectively. This radiolucent area increased slowly thereafter, but not statistically significantly, up to 56 days (Fig. 1b). Quantitative µCT analysis of the lesion (i.e. apical radiolucent area) volume showed a significant difference between the animals without exposure (W/O) and those with exposure after 28 days. In addition, inflammatory cell infiltration at the AP was clearly increased after 28 days (Fig. 1c) indicating the development of AP and consistent with previous findings^47,48^. We then performed immunostaining to investigate the extent of neutrophil infiltration at the periapical area following pulp exposure. Infiltration by Ly6g (a neutrophil marker) and MMP9 (an inflammatory marker) were observed until 14 days but these cells had disappeared by 28 days (Fig. 1d). During the maturation stage of the apical lesion, anti-Mac2 positive macrophages were detectable at 14 days after the exposure and could be observed at remarkably increased levels after 28 days (Fig. 1e). These results indicated that a neutrophil-based inflammatory response was active as an early process at 14 days and a macrophage-based reaction was functioning at 28 days after exposure. These results further suggested that an AP-initiated innate immune response against bacterial infection induces neutrophil infiltration at the initial stages and that macrophages are subsequently recruited during the development of the lesion.

### Identification of CXCL9 as a major chemokine expressed in macrophages in the AP mouse model

**Figure 2:**
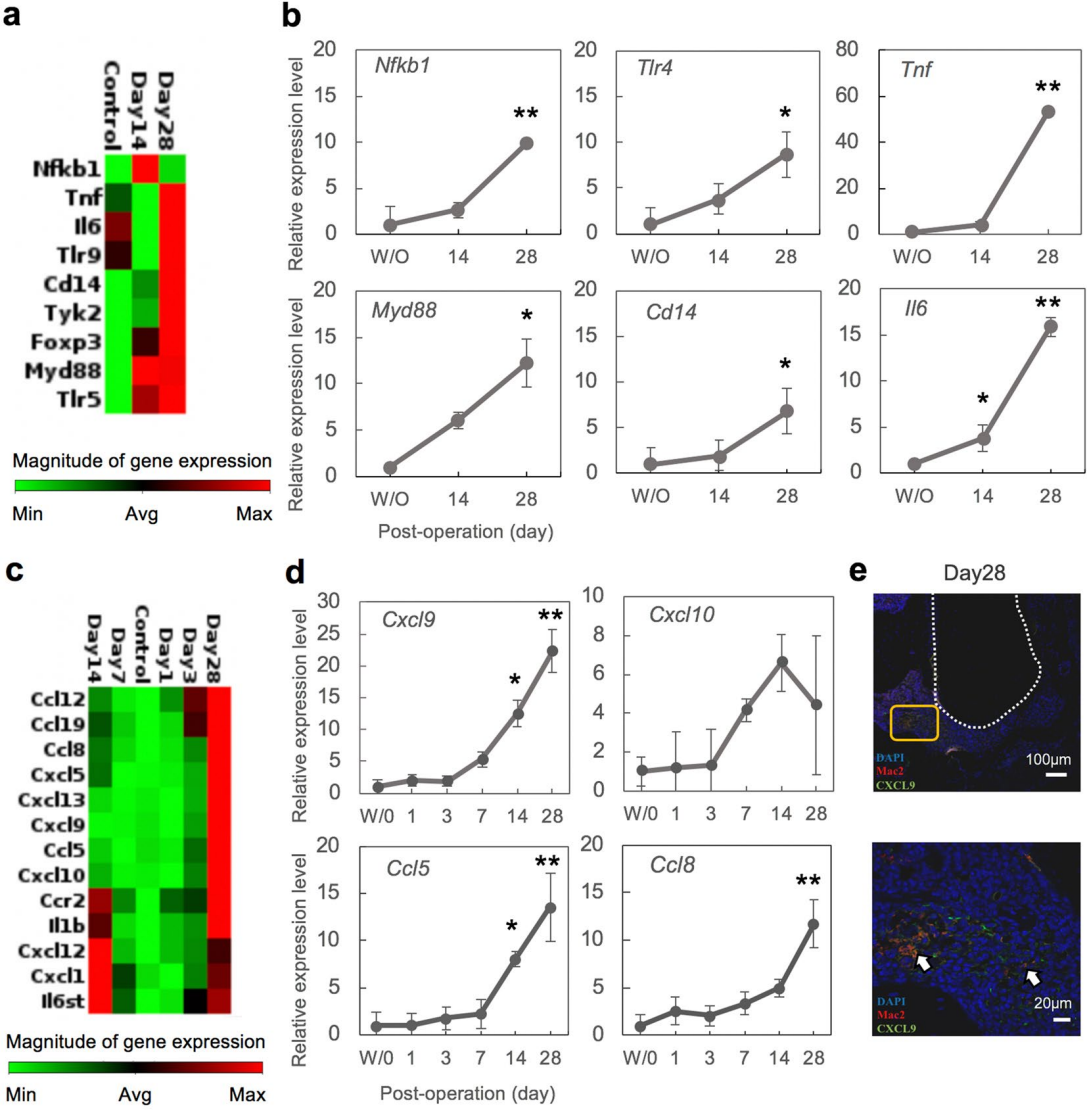
Inflammatory markers especially chemokines are highly expressed in periapical lesions. (**a**) Clustergram analysis of the gene expression patterns for inflammation-associated factors during the development of AP (n = 1 per group). (**b**) Validation of the inflammation-associated factors by quantitative PCR analysis. Normalization was based on the GAPDH expression in the without (W/O) exposure group. Statistical analysis was performed using the Dunnett test in comparison with the W/O group; *p < 0.05, **p < 0.01 (mean ± SD, n = 4 per group). (**c**) Clustergram analysis of inflammatory mediator and receptor genes identified during the development of AP (n = 1 per group). (**d**) Validation of the inflammatory markers by quantitative PCR analysis. Statistical analysis was performed using the Dunnett test in comparison with the W/O group; *p < 0.05, **p < 0.01 (mean ± SD, n = 4 per group). (**e**) Representative images of immunostained CXCL9- and Mac2-positive macrophages (arrows) at 28 days after pulp exposure. The lower panel shows a higher magnification image of the framed region in the upper panel.

We next conducted global gene expression analysis of the innate and adaptive immune systems in our AP model using an RT^2^ PCR Array (PAMM-052ZR) to determine whether macrophage-based inflammation was involved in the development of AP. The results of our PCR Array analysis of inflammation-associated factors indicated an increased expression of inflammatory markers related to toll-like receptor signaling such as *Nfkb1, Myd88, Tlr4, Cd14*, and cytokines such as *Tnf* and *Il6*, until 28 days after exposure (Fig. 2a,b). These data indicated that macrophage migration accompanied by toll-like receptor signaling is involved in the development of periapical lesions. Hence, we speculated that the molecules which regulate macrophage migration and activation, such as chemokines and cytokines, affect periapical lesion formation.

To test this hypothesis, we performed PCR Array (PAMM-011Z) analysis of inflammatory cytokines and receptors. In addition to inflammatory markers including *Il1b*, chemokines have also been shown to be highly expressed after 14 days of left mandibular first molar pulp exposure (sFig. 3). Among these factors, we found a gene cluster of chemokines such as *Ccl 5, 8, 12, 19, Cxcl 1, 5, 9,10, 12, 13* and *Ccr2* which was highly expressed at day 28, while no expression of anti-inflammatory cytokines such as IL-10 and TGFβ was observed. Interestingly, the expression pattern of these genes was almost identical to that of *Il1b*, which has been shown as a critical factor for AP^43^, suggesting that these genes could play a role in AP development (Fig. 2c). We then further confirmed the expression of these molecules using quantitative PCR analysis. Among these factors, CXCR3- and CCR3-related chemokines such as *Cxcl9*, *Ccl5* and *8* became increased at day 7 and were found to be further elevated at days 14 and 28 (Fig. 2d). *Cxcl10* was also increased at days 7 and 14 but decreased at day 28. *Cxcl9* was more highly expressed than the others and we performed immunohistochemical analysis to investigate the localization of these chemokines at periapical tissues. As shown in Fig. 2e, macrophage-derived CXCL9 and Mac2-positive macrophages were observed, suggesting that macrophages expressing CXCL9 are recruited during the onset of periapical lesions. Anti-inflammatory cytokines such as IL-10 and TGFβ were not detected (data not shown) whereas FoxP3, a marker for regulatory T cells, was found to be upregulated but quantitative PCR analysis did not confirm its expression pattern. We therefore did not investigate these genes further in our present analyses. The onset of AP was investigated up to 28 days in this study, and detailed analyses are required for more than 28 days to understand the expression of other inflammatory markers.

### A CXCR3 antagonist reduces the AP lesion volume

To investigate whether CXCL9 plays a role in periapical lesion formation, an antagonist of this cytokine was administrated into the mouse AP model via oral gavage and evaluated by µCT and histological analysis. SCH546738 is a small molecule non-competitive CXCR3 antagonist with a far higher affinity than AMG487 and binds to CXCR3 at an allosteric site and changes its conformation^49^.

**Figure 3:**
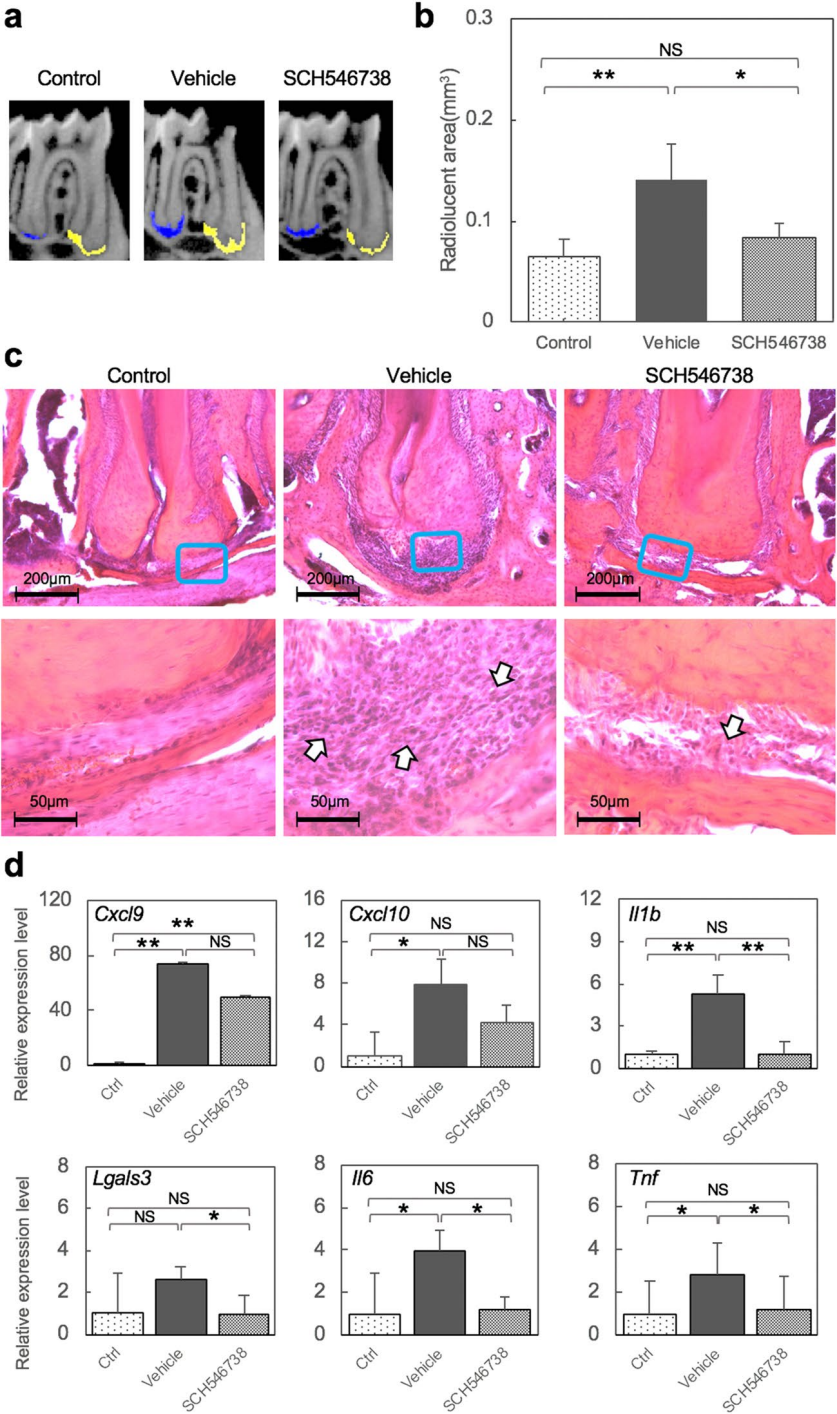
An orally administered CXCR3 antagonist suppresses periapical lesion formation. (**a**) Representative μCT images of the AP mouse at 28 days after pulp exposure. The radiolucent area is indicated by yellow and blue colors, determined using TRI/3D-BON software based on the CT values. (**b**) Quantitative analysis of the radiolucent area (mm^3^) from the μCT images. Statistical analysis was conducted using the Turkey-Kramer test; *p < 0.05, **p < 0.01 (mean ± SD, n = 5 per group). (**c**) HE staining around the periapical area at 28 days after pulp exposure. The arrow highlights inflammatory cell filtration. (**d**) Validation of the inflammatory markers by quantitative PCR analysis. Statistical analysis was conducted using the Turkey-Kramer test; *p < 0.05, **p < 0.01 (mean ± SD, n = 4 per group).

**Figure 4:**
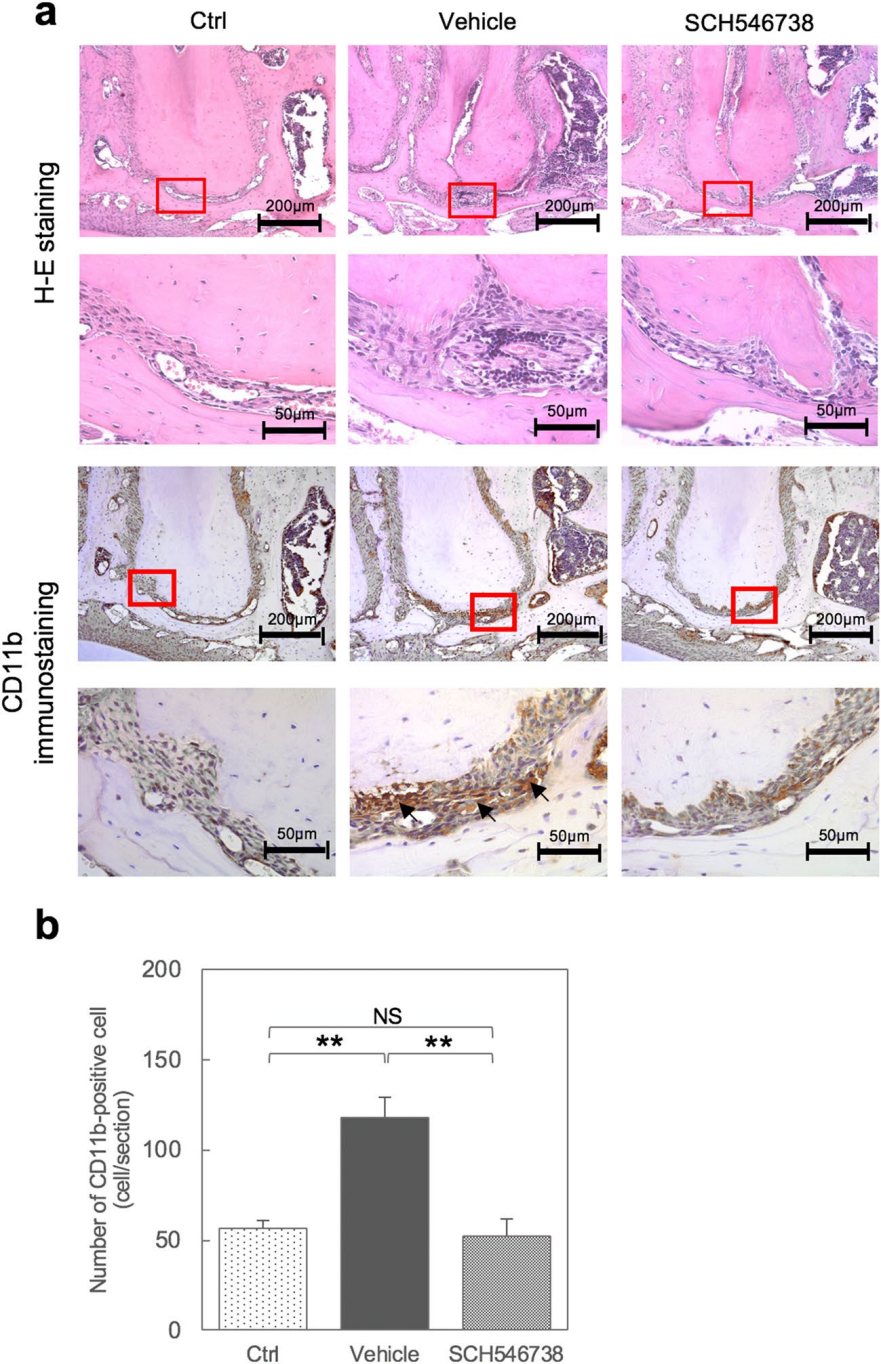
A CXCR3 antagonist reduces CD11b inflammatory cell infiltration in the mouse AP model. (**a**) HE staining and immunostaining of CD11b-positive inflammatory cells (arrows) at 28 days after pulp exposure to SCH546738, vehicle or control with a lower magnification (upper panel) and higher magnification (lower panel) of the square area shown. (**b**) Quantitative analysis of CD11b-stained cells demonstrated significant immune cell infiltration in the vehicle sample compared with the control and SCH543738 treatment groups. Data are expressed as a mean ± S.D; * p < 0.05 compared with the control group.

Sagittal sections of µCT images revealed smaller periapical lesion formation at the apex of the mesial and distal roots in the SCH546738 treatment group (Fig. 3a). Quantitative analysis indicated that this CXCR3 antagonist significantly inhibited lesion formation compared with vehicle (Fig. 3b). HE staining analysis further revealed that bone resorption at periapical tissues was reduced in CXCR3 antagonist treatment group compared with the vehicle control. A higher magnification view confirmed that CXCR3 antagonist administration resulted in fewer inflammatory cell infiltrations (arrows) in the periapical lesion compared with the vehicle (Fig. 3c). Quantitative PCR analysis indicated that the SCH546738 treatment did not affect the expression of *Cxcl9* or *10* but that a significant reduction of inflammatory cytokines such as *Il1b, Il6* and *Tnf* was also caused by this antagonist (Fig. 3d). HE staining showed fewer inflammatory cell infiltration and immunohistochemical analysis confirmed that fewer CD11b-positive inflammatory cells infiltrations were evident in the SCH546738 treatment group (Fig. 4a). In contrast, neither inflammatory cell infiltration nor bone resorption were affected in the control group. Quantitative analysis of CD11b-positive cells confirmed that the CXCR3 antagonist significantly inhibited inflammatory cell migration compared with the vehicle treatment (Fig. 4b). Taken together, these results indicated that a CXCL9-CXCR3 axis plays an important functional role in the onset of periapical lesions through the activation of macrophages.

### CXCL9 induces macrophage migration and activation

**Figure 5:**
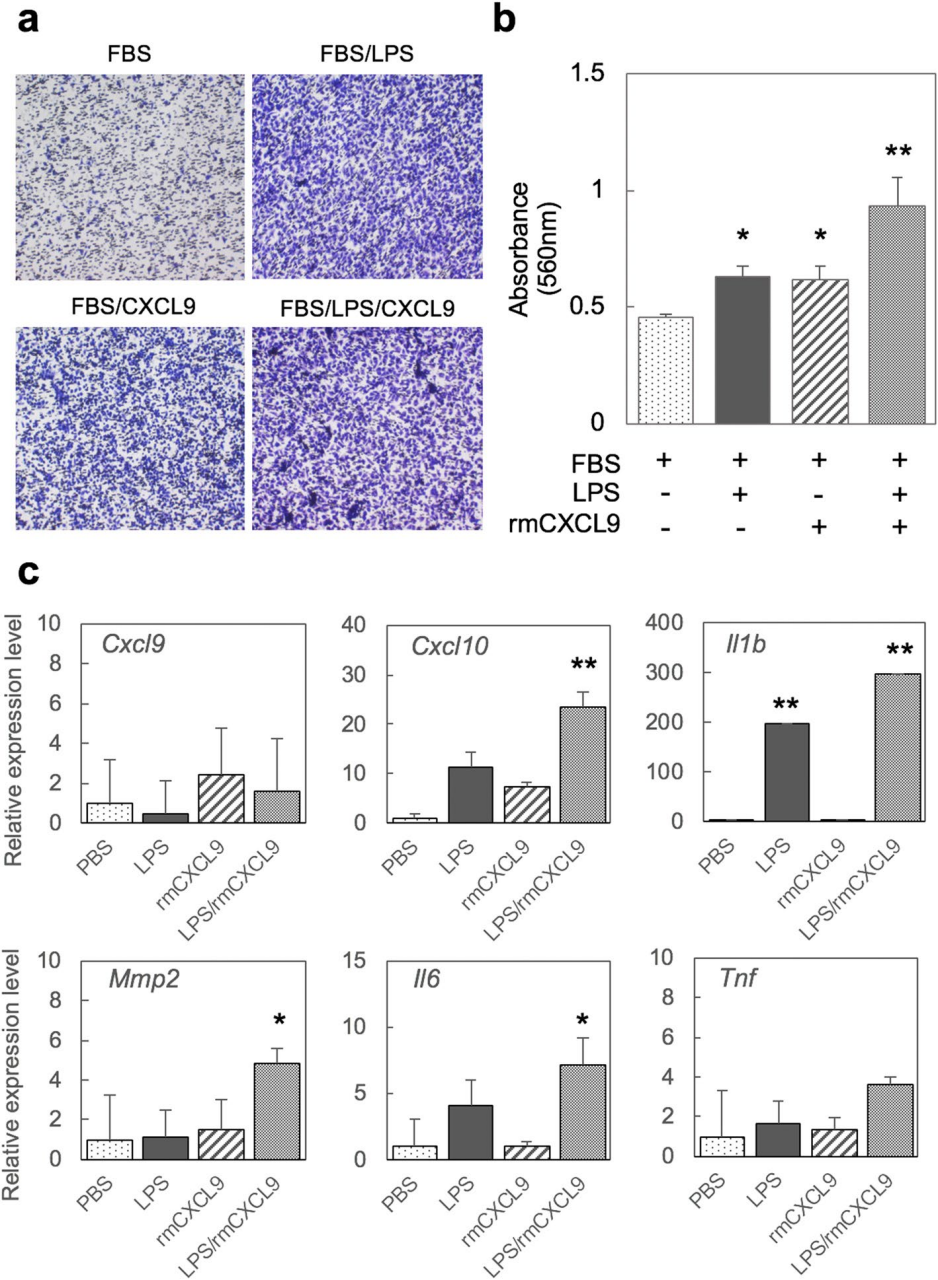
CXCL9 induces macrophage migration and enhances the inflammatory response to LPS. (**a**) Representative images of peritoneal macrophages that have moved through the membrane. Peritoneal macrophages were stained using a commercial solution in accordance with the manufacturer’s protocol. (**b**) Cell migration assay for 24 h was analyzed using the colorimetric absorbance at 560 nm (mean ± SD, n = 4 per each group). (**c**) Validation of the inflammatory markers by quantitative PCR analysis. Statistical analysis was conducted using the Dunnett test to compare with the PBS group; *p < 0.05, **p < 0.01 (mean ± SD, n = 4 per group).

We further evaluate the role of CXCL9 in the migration and activation of macrophages using mouse peritoneal macrophages and a human monocytic leukemia cell line (THP-1). A chemotaxis assay was first performed to evaluate macrophage migration following exposure to rmCXCL9. The results indicated that macrophages had migrated across the membrane under the influence of rmCXCL9 in a similar manner to those in a positive-control LPS group (Fig. 5a). This migration ability was enhanced by the combination of rmCXCL9 and LPS treatments (Fig. 5b). We next performed a macrophage inflammatory assay using quantitative PCR to evaluate the role of CXCL9 in the activation of these immune cells. Quantitative PCR analysis revealed that an rmCXCL9 single treatment had no obvious effects on the expression levels of inflammatory markers such as *Cxcl9, 10, Il1b, Il6, Tnf* and *Mmp2*. Notably however, *Cxcl10, Il1b, Mmp2* and *Il6* showed high expression upon exposure to a combination of rmCXCL9 and LPS (Fig. 5c). The macrophage-activation effects of rmCXCL9 were confirmed in the THP-1 cell line in which inflammatory markers such as *CXCL9, IL1B, IL6, TNF,* and *MMP2* are significantly induced. The expression of these markers was inhibited by treatment with SCH546738, a CXCR3 antagonist.

To next investigate the regulation of macrophage activation via the CXCL9-CXCR3 axis, we applied rmCXCL9 and the CXCR3 antagonist to THP-1 cells after Phorbol12-myristate13-acetate (PMA) treatment. The results supported a regulatory role of CXCL9 in the migration of macrophages and in lesion development in AP (sFig. 4). Quantitative PCR analysis indicated that treatment of the THP-1 cells with rmCXCL9 upregulated the inflammatory markers associated with macrophages such as *IL1B*, *IL6, TNF* and *MMP2*. These markers were in turn suppressed by the CXCR3 antagonist.

**Figure 6:**
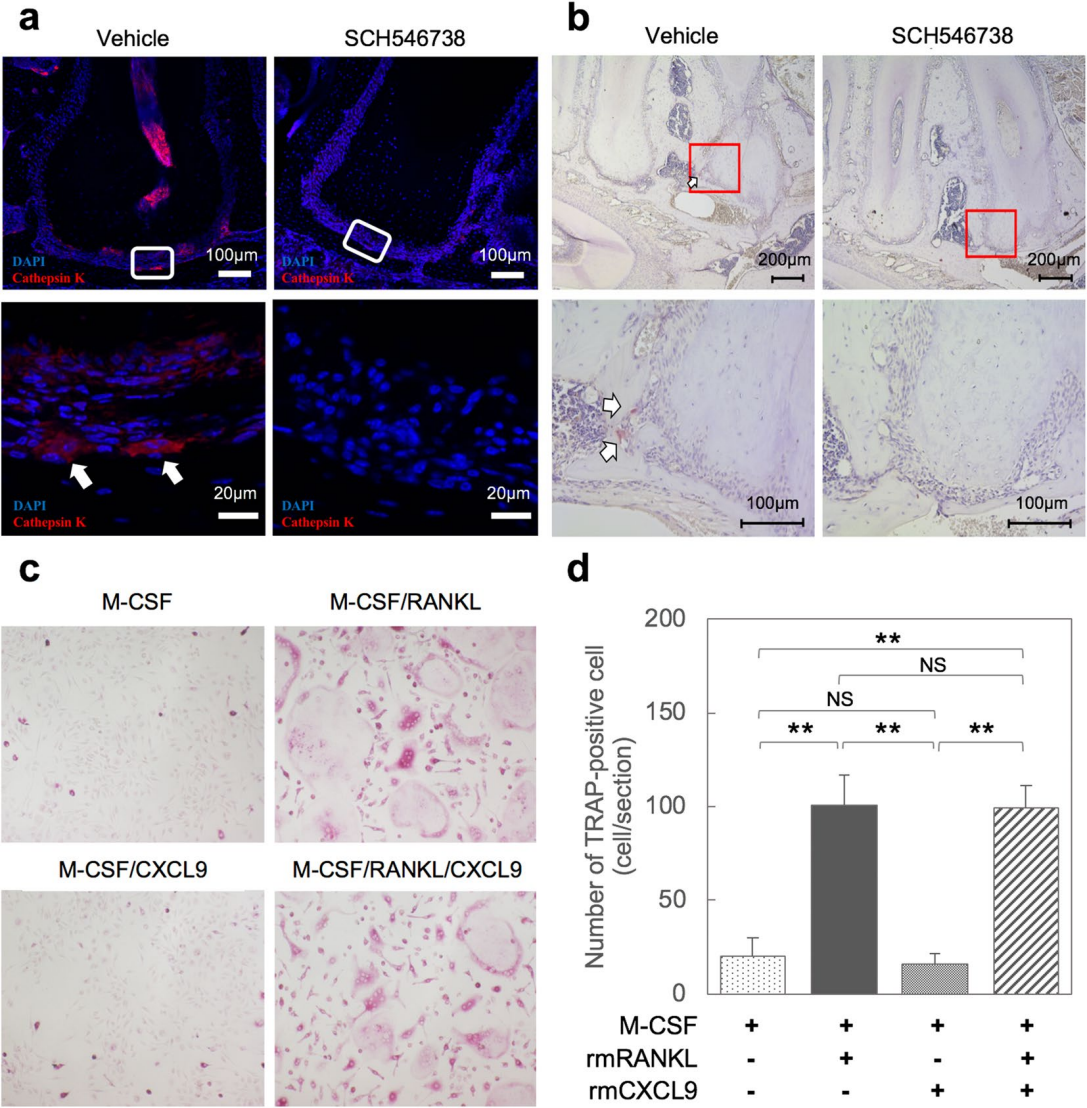
The CXCL9-CXCR3 axis is involved in the regulation of osteoclast activity but does not directly affect RANKL-induced osteoclast formation. (**a**) Representative immunostaining images of cathepsin K-positive inflammatory cells (arrows) at 28 days after pulp exposure in the AP mouse model. (**b**) TRAP staining around the periapical area at 28 days after pulp exposure. The arrow denotes TRAP-positive multinuclear cells. (**c**) Microscopic images of TRAP-positive cells. Osteoclast precursors were treated for 5 days with (i) Macrophage colony stimulating factor (M-CSF) alone; (ii) M-CSF and RANKL; (iii) M-CSF, RANKL and CXCL9; or (iv) M-CSF and CXCL9. (**d**) Numbers of TRAP-positive cells (cell/section). For quantification of TRAP positive cells, we selected 5 independent areas per one well and quantified using ImageJ software. Statistical analysis was conducted using he Turkey-Kramer test; *p < 0.05, **p < 0.01 (mean ± SD, 20 areas/n = 4 per group).

Finally, we functionally tested whether rmCXCL9 directed the differentiation of macrophages into osteoclasts. Immunostaining of cathepsin K (Fig. 6a) or TRAP (Fig. 6b) staining revealed an inhibition of osteoclast recruitment in the periapical lesion upon exposure to the SCH546738 CXCR3 antagonist. An osteoclast differentiation assay using M-CSF dependent bone marrow macrophages (MDBMs) indicated an absence of TRAP-positive multinucleated like cells in the M-CSF/CXCL9 group similar to that of treatment with M-CSF only (negative control) (Fig. 6c). In contrast, multinucleated cells were clearly evident when MBDMs were stimulated with M-CSF/RANKL (positive control), but this was similar to cells treated with M-CSF/RANKL/CXCL9 (Fig. 6c). Quantitative analysis subsequently indicated that TRAP-positive osteoclasts were not increased upon M-CSF/RANKL/CXCL9 treatment compared to M-CSF/ RANKL exposure, suggesting that CXCL9 is recruited and activated macrophages to induce osteoclastogenic cytokines and promote the differentiation of osteoclast precursor cells into osteoclasts (Fig. 6d).

We next investigated the expression of CXCR3 during osteoclast differentiation. The results showed that osteoclast markers such as cathepsin K (*Ctsk)*, calcitonin receptor (*Calcr)*, nuclear factor of activated T-cells (*Nfatc1)*, osteoclast stimulatory transmembrane protein (*Oc-stamp)* and matrix metalloproteinase 9 (*Mmp9)* become highly expressed after 3 days of culture, but the relative expression of *Cxcr3* (6 >) was lower compared with those of *Ctsk* (200 >), *Calcr* (60 >), *Nfatc1(25* >), *OC-Stamp(20* >) and *Mmp9* (700 >) (sFig. 5).

Following the confirmation of CXCR3 expression in osteoclast progenitors, we next investigated the effects of rmCXCL9 on osteoclast differentiation at 0, 3, and 5 days. The results showed that the number of osteoclasts cultured in M-CSF/RANKL did not increase upon treatment with rmCXCL9 at 0, 3 and 5 days (sFig. 6a,b). These data suggested that CXCL9 is recruited and activates macrophages to induce osteoclastogenic cytokines and promote the differentiation of osteoclast precursor cells into osteoclasts (Fig. 6d).

## Discussion

**Figure 7:**
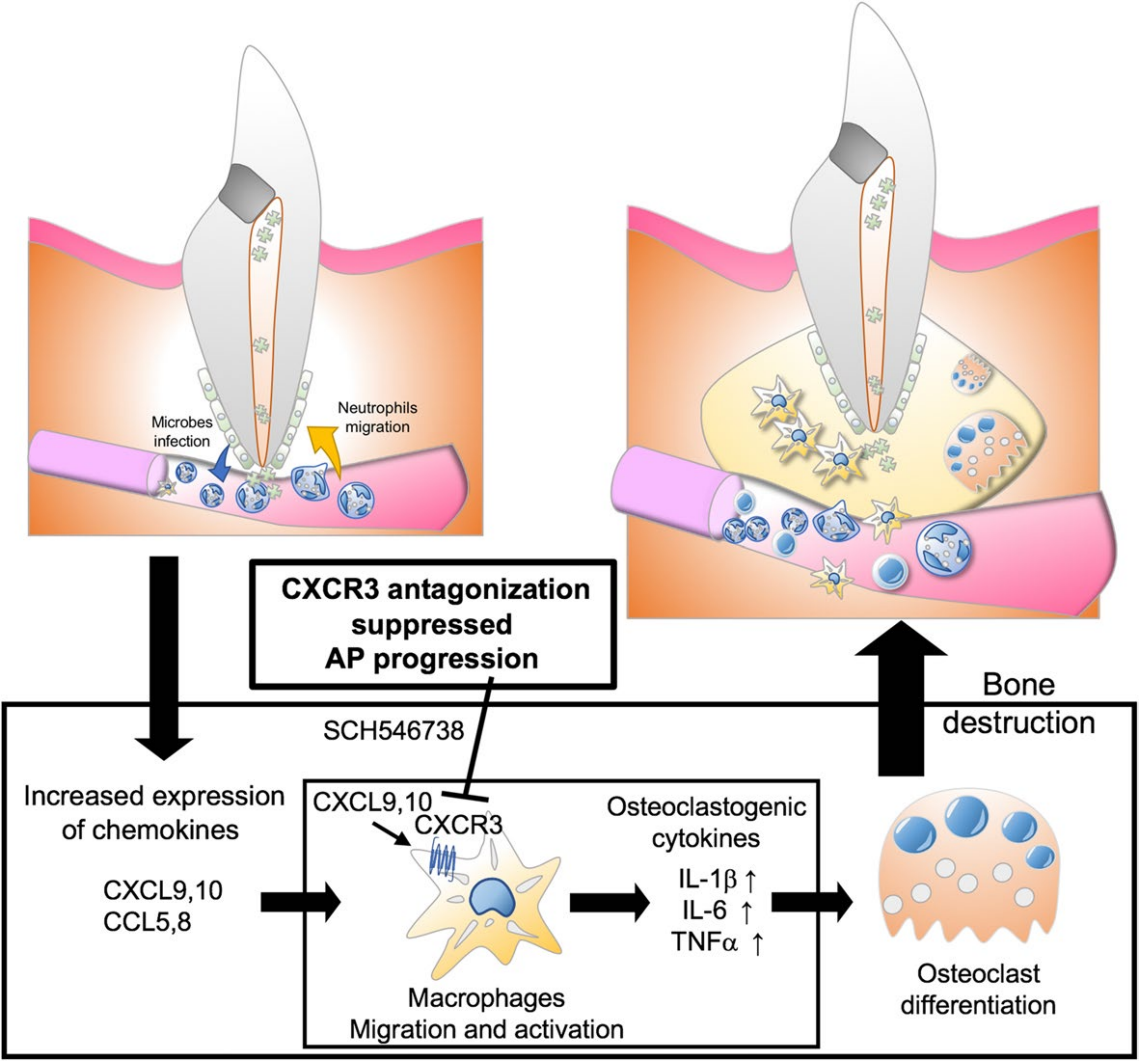
Model for the inflammatory mechanism mediated by the CXCL9,10—CXCR3 axis during AP progression. The production of CXCL9 and10 and their receptor CXCR3 is induced by bacterial infection in the root canal. These molecules induce chemotaxis, promote the differentiation of macrophages, and produce osteoclastogenic cytokines to promote bone destruction by activating osteoclasts during the development of AP. A CXCR3 blockade suppresses the tissue destruction caused by an AP lesion through the inhibition of macrophage activation and migration.

AP is a chronic inflammatory disease caused by oral bacterial infection. AP is primarily treated by disinfection of the root canal, which is carried out to reduce the immune and inflammatory reactions and thereby promote wound healing of the periapical tissue^1–5^. Anti-inflammatory approaches represent an alternative strategy to promote healing in therapy-resistant AP cases. A mouse AP model has been extensively used to investigate the molecular pathogenesis of this disease, the effects of medications, and the systemic conditions underlying the development of AP^42,43,47,48^. In our current study, we used the AP mouse model to screen for inflammatory-related molecules and identify highly expressed markers during AP development. We found from our analyses that CXCL9, a small cytokine belonging to the CXC chemokine family, and its receptor CXCR3, play a role in chemotaxis induction and macrophage activation, processes which in turn recruit and activate osteoclasts during AP development. Notably, the blocking of CXCR3 using an antagonist suppressed AP development through the inhibition of macrophage activation and migration. Thus, our current data provide new insights into the future development of anti-inflammatory therapeutic regimens targeting the CXCL9-CXCR3 axis that promote accelerated healing and reduce inflammatory bone destruction in cases of AP (Fig. 7).

A growing body of literature has indicated that significant efforts have been made using mouse models to better understand and characterize the signaling pathways in inflammatory bone resorption in AP, and also the cells involved in this disorder^42,43,47,48^. It has been suggested that toll like receptors (TLR) are crucial factors in the activation of the innate immune system in response to primary endodontic infections. A prior study by Daniel Rider et al. reported that TLR2-deficient mice show progression of AP with increasing CD14-positive macrophages, whereas a TLR2/4 double deficiency rescued the progression of AP in the mice, indicating that multiple TLR and innate immune cells are involved in the regulation of AP^48^. Additionally, various studies have proposed that TLR signaling promotes the induction of proinflammatory cytokines such as IL-1β, IL-6 and TNFα that are highly expressed in both an experimentally induced mice AP model and in human periapical lesions that promote bone destruction^17,18,42,43,47,48^. In our present study, we confirmed that TLR signaling-related genes, including *Tlr4, Nfkb, Myd88, Cd14, Il6* and *Tnf*, are gradually increased in the AP mouse model following induction of the infection and become highly expressed when lesion formation accompanied by bone destruction is established. Our present results have indicated that bacterial infection from an infected root canal stimulates the recruitment of innate immune cells such as macrophages through the activation of TLR signaling, which leads to a progression of AP. A number of studies investigating the pathogenesis of AP in mouse models have identified an influx of neutrophils at an early stage and that macrophages along with osteoclasts are recruited to the periapical area and cause bone destruction as the lesion progress^42,43,47,48^. Similarly, our current immunobiological data confirmed that neutrophil recruitment was clearly evident during the early immune responses to AP, as a result of the high expression of chemokines (*Cxcl15, Ccl1, 3, 4, 9, 11, 22, 24*) and inflammatory cytokines (*Tnf, Il3,4,5,7,13,11,16,17α,17β*). As the lesion progressed, we detected the dominant expression of specific chemokines such as *Cxcl9, Cxcl10, Ccl5* and *Ccl8*, which can recruit macrophages, and clear evidence of osteoclast presentation in the lesion area. Macrophages are important phagocytic cells in the innate immune system and secrete proinflammatory cytokines such as IL-1β, IL-6, TNFα to induce the differentiation of osteoclast precursors derived from circulating macrophages. Our findings indicate that specific chemokines involved in macrophage recruitment and activation underlie the eventual progression in AP to tissue destruction. A previous study has reported in this regard that that inhibition of macrophage activation using an anti-CD14 antibody suppressed the AP lesion size in TLR2 KO mice^48^. These findings collectively support the notion that the migration and activation of macrophages induced by chemokines such as CXCL9 contribute to AP lesion formation and eventual alveolar bone destruction via osteoclast recruitment and activation.

Recent evidence has suggested a strong correlation between the expression of chemokines and chemokine-induced macrophage recruitment in inflammatory conditions^29–32^. A number of studies have confirmed the presence of various chemokine receptors such as CCR1, CCR2, CCR3, CCR5, CXCR1, CXCR3 and chemokines such as CCL2, CCL5, CXCL10, CXCL12 in AP lesions from both humans and mice^38,39^. In our present study, we found that numerous chemokines and their receptors are expressed during the development of AP. However, specific chemokines such as CXCL9, CXCL10 and their receptor CXCR3 were found to be involved in macrophage recruitment and activation in our current mouse AP model experiments. It has also been proposed that the axis of CXCL9, CXCL10 and CXCR3 functions primarily in immune cell migration, differentiation, and in the activation of cytotoxic T cells, natural killer T cells and macrophages. The authors of that study concluded that the CXCL9, CXCL10 and CXCR3 expression induced by IFNγ in M1 macrophages causes the expression of inflammatory cytokines including IL-1β, IL-6 and TNFα and results in the progression of tissue destruction in various disorders such as metabolic diseases, asthma, allergies, atherosclerosis, fibrosis, reduced wound healing, and autoimmunity^50^. Indeed, it has been reported also that CXCL9 and CXCL10 act as macrophage chemoattractants in arthritis, chronic cardiac inflammation, and cancer, thus implicating these chemokines in inflammatory disease^34–37^. In addition, the accumulation and activation of tissue resident macrophages, such as perivascular macrophages, has been shown to be dependent on CXCR3 and CXCL10 during the arterial remodeling triggered by hemodynamic stress^51^. In contrast with earlier findings, another study reported that CXCR3 signaling suppress M2 macrophage polarization in breast cancer tumors^52^. From this, we speculate that the activation of M1 macrophages by CXCR3 signaling promotes the inflammatory cytokine expression that plays a role in the onset of AP. Further studies are needed however to more precisely elucidate the detailed mechanisms by which the CXCL9, CXCL10 and CXCR3 axis controls macrophage recruitment and activation.

Our current cluster gene analysis and quantitative PCR data indicated a high expression of the cytokines *Il1b, Il6, Tnf* and chemokines *Cxcl9, Cxcl10, Ccl5* and *Ccl8,* in parallel with the presence of macrophages. These macrophages were possibly of an M1 phenotype as they were found to be associated with pro-inflammatory cytokines. In addition, our current in vitro data revealed an enhanced migration of macrophages under stimulation by both CXCL9 and LPS. Although the responses of THP-1 and peritoneal macrophages to CXCL9 were not identical, both of these cell types showed a high expression of *Il1b, Il6* and *Tnf* by quantitative PCR analysis, which was consistent with our in vivo data. These data collectively suggest that the CXCL9-CXCR3 axis contributes to the macrophage migration and activation that induce the expression of pro-inflammatory cytokines necessary for AP progression and development. Much work has been conducted to date on the potential impact of chemokines on osteoclast activation and the important role of this in the lesion formation and bone destruction that occurs in AP^45,53^. More recent evidence has demonstrated increased levels of CXCL8, CXCL9, CXCL10, and CCL20 in rheumatoid arthritis, indicating the importance of chemokines in inflammatory bone diseases^53^. Among these factors, CXCL10 indirectly induces osteoclast genesis by promoting RANKL and TNFα expression in activated CD4^+^ T cells. Reciprocally, RANKL also induces CXCL10 expression in osteoclast precursors. In addition, anti-CXCL10 antibodies suppress bone destruction in a collagen-induced arthritis model^36^. Similarly, we found in our present experiments that CXCL9 does not directly promote osteoclast differentiation but activates macrophages which secrete the cytokines (IL-1β, IL-6 and TNFα) that can stimulate osteoclastogenic associated bone loss. It has been well established that RANKL is induced in AP models and our current model showed this expression pattern also. Although we need to further investigate whether CXCL9 accelerates LPS induced osteoclastgenesis^54^, our current data strongly suggest that a CXCL9-CXCR3 axis enhances osteoclastogenesis via specific cytokines released from activated macrophages.

Considering the complex interactions involving CXCL9 secreted from immune cells as a result of root canal bacteria that regulate the functions of the macrophages responsible for periapical inflammation and bone destruction, we investigated the effect of CXCL9 and its mechanism of action in the pathogenesis of AP. The abundant expression of CXCL9 during the development of AP make it a potential therapeutic target. Our mouse AP model was treated with a CXCR3 antagonist that blocks the activity of CXCL9,10 and 11 and we then evaluated the expression of the macrophage-derived proinflammatory cytokines involved in bone resorption. The CXCR3 antagonist decreased the levels of the inflammatory cytokines produced by macrophages, indicating the importance of this chemokine as a target molecule in the protective immune response. The inhibition of periapical lesions by the CXCR3 antagonist possibly modulated inflammatory cytokine expression in these cells, which in turn prevented bone destruction by inhibiting matrix metalloproteinase and recruiting osteoclasts. Indeed, CXCR3 antagonism has been associated with inflammatory and autoimmune diseases such as rheumatoid arthritis, multiple sclerosis, inflammatory bowel disease, systemic lupus erythematosus, chronic obstructive pulmonary disease, psoriasis, organ transplant rejection and graft-vs-host disease, asthma, and type 1 diabetes^50^. CXCR3 is predominantly expressed on Th1 CD4^+^ T cells and CD8^+^ cytotoxic T cells, and activates signaling molecules including Gαi protein and β-arrestin that can initiate intracellular signaling events, cell migration and proinflammatory cytokine production^50^. Hence, the CXCR3 receptor is a target molecule for the treatment of various inflammatory diseases. Our present data confirm that the progression of inflammatory bone destruction induced by oral bacterial infection can be suppressed by antagonizing CXCR3. A recent study has also demonstrated that the progression of periodontal disease, an inflammatory disease similar to AP, was inhibited by a blockade of CXCR3 in both deficient mice, and via treatment with the AMG487 antagonist^55^. In our present study, we used SCH546738, a CXCR3 chemokine antagonist similar to AMG487, which is approved by the FDA and has a well demonstrated efficacy in various preclinical models of inflammatory diseases^49^. Our results confirmed that SCH546738 significantly reduces the extent of the apical tissue destruction in an AP mouse model by decreasing the levels of inflammatory cytokines, and hence macrophage recruitment and activation, resulting in reduced osteoclast activation. A recent study has reported faster healing of periapical tissues when a TNFα antibody was used as an anti-inflammatory therapy in inflammatory bowel disease patients, who are considered to be at high risk of therapy-resistant AP^12^. These results strongly suggest that anti-inflammatory approaches involving a blockade of chemokine receptors such as CXCR3 would be a beneficial approach to treating therapy resistant AP. In this study, we only investigated the effect of CXCR3 antagonist on systemic administration but this approach is not appropriate for human translation. Further studies are necessary to develop a local drug delivery system into AP lesion for clinical translation.

In conclusion, our current findings provide new insights into the role of CXCL9 in the macrophage responses and functions in AP and suggest that an intervention involving a pharmacologic blockade of CXCL9 activity is a potential therapeutic approach for therapy resistant AP. CXCR3 antagonists can significantly suppress proinflammatory cytokines during the pathogenesis of AP and have the potential to reduce the periapical lesion size in this disease. CXCL9-induced macrophage migration and activation promote inflammatory cytokine expression, which in turn activates osteoclasts and promotes bone resorption. The acquired/adaptive immune responses play an important role in the development of therapy resistant AP in patients with other systemic diseases, whilst the innate immune response is mainly responsible in the mouse AP model, as shown in our present study. Further studies are necessary to determine the relationship between AP and systemic disease and to investigate whether the acquired immune system accelerates severe tissue destruction in AP.

## Supplementary Information

Supplementary Information.

## References

[1] Stashenko P (1990). Role of immune cytokines in the pathogenesis of periapical lesions. Endod. Dent. Traumatol..

[2] Siqueira JF, Rocas IN (2008). Clinical implications and microbiology of bacterial persistence after treatment procedures. J. Endod..

[3] Jonsson D, Nebel D, Bratthall G, Nilsson BO (2011). The human periodontal ligament cell: A fibroblast-like cell acting as an immune cell. J. Periodontal Res..

[4] Darveau RP (2009). The oral microbial consortium's interaction with the periodontal innate defense system. DNA Cell Biol..

[5] Colic M (2009). Proinflammatory and immunoregulatory mechanisms in periapical lesions. Mol. Immunol..

[6] Weber M (2019). Differences in inflammation and bone resorption between apical granulomas, radicular cysts, and dentigerous cysts. J. Endod..

[7] Ricucci D, Siqueira JF, Bate AL, Pitt Ford TR (2009). Histologic investigation of root canal-treated teeth with apical periodontitis: A retrospective study from twenty-four patients. J. Endod..

[8] Nair PN, Sjogren U, Krey G, Kahnberg KE, Sundqvist G (1990). Intraradicular bacteria and fungi in root-filled, asymptomatic human teeth with therapy-resistant periapical lesions: A long-term light and electron microscopic follow-up study. J. Endod..

[9] Virtanen E (2017). Apical periodontitis associates with cardiovascular diseases: A cross-sectional study from Sweden. BMC Oral Health.

[10] Segura-Egea JJ (2005). High prevalence of apical periodontitis amongst type 2 diabetic patients. Int. Endod. J..

[11] Kohsaka T, Kumazawa M, Yamasaki M, Nakamura H (1996). Periapical lesions in rats with streptozotocin-induced diabetes. J. Endod..

[12] Poyato-Borrego M (2020). High prevalence of apical periodontitis in patients with inflammatory bowel disease: An age- and gender-matched case-control study. Inflamm. Bowel Dis..

[13] Segura-Egea JJ, Martin-Gonzalez J, Castellanos-Cosano L (2015). Endodontic medicine: Connections between apical periodontitis and systemic diseases. Int. Endod. J..

[14] Silva TA (2005). Differential expression of chemokines and chemokine receptors in inflammatory periapical diseases. Oral Microbiol. Immunol..

[15] Braz-Silva PH (2019). Inflammatory profile of chronic apical periodontitis: A literature review. Acta Odontol. Scand..

[16] Graves DT, Oates T, Garlet GP (2011). Review of osteoimmunology and the host response in endodontic and periodontal lesions. J. Oral Microbiol..

[17] Hahn CL, Liewehr FR (2007). Innate immune responses of the dental pulp to caries. J. Endod..

[18] Martinho FC (2014). Signaling pathways activation by primary endodontic infectious contents and production of inflammatory mediators. J. Endod..

[19] Shapouri-Moghaddam A (2018). Macrophage plasticity, polarization, and function in health and disease. J. Cell Physiol..

[20] Davies LC, Jenkins SJ, Allen JE, Taylor PR (2013). Tissue-resident macrophages. Nat. Immunol..

[21] Italiani P, Boraschi D (2014). From monocytes to M1/M2 macrophages: Phenotypical vs. functional differentiation. Front. Immunol..

[22] Weber M (2018). Macrophage polarization differs between apical granulomas, radicular cysts, and dentigerous cysts. Clin. Oral Investig..

[23] Metzger Z (2009). Healing kinetics of periapical lesions enhanced by the apexum procedure: A clinical trial. J. Endod..

[24] Pereira M (2018). Common signalling pathways in macrophage and osteoclast multinucleation. J. Cell Sci..

[25] Teixeira-Salum TB (2010). Distinct Th1, Th2 and Treg cytokines balance in chronic periapical granulomas and radicular cysts. J. Oral Pathol. Med..

[26] Haringman JJ, Kraan MC, Smeets TJ, Zwinderman KH, Tak PP (2003). Chemokine blockade and chronic inflammatory disease: Proof of concept in patients with rheumatoid arthritis. Ann. Rheum. Dis..

[27] Raman D, Sobolik-Delmaire T, Richmond A (2011). Chemokines in health and disease. Exp. Cell Res..

[28] Rossi D, Zlotnik A (2000). The biology of chemokines and their receptors. Annu. Rev. Immunol..

[29] Mosser DM, Edwards JP (2008). Exploring the full spectrum of macrophage activation. Nat. Rev. Immunol..

[30] Karlmark KR (2010). The fractalkine receptor CX(3)CR1 protects against liver fibrosis by controlling differentiation and survival of infiltrating hepatic monocytes. Hepatology.

[31] Scheuerer B (2000). The CXC-chemokine platelet factor 4 promotes monocyte survival and induces monocyte differentiation into macrophages. Blood.

[32] Mazzon C (2016). CCRL2 regulates M1/M2 polarization during EAE recovery phase. J. Leukoc. Biol..

[33] Schulthess FT (2009). CXCL10 impairs beta cell function and viability in diabetes through TLR4 signaling. Cell Metab..

[34] House IG (2020). Macrophage-derived CXCL9 and CXCL10 are required for antitumor immune responses following immune checkpoint blockade. Clin. Cancer Res..

[35] Hardison JL, Wrightsman RA, Carpenter PM, Lane TE, Manning JE (2006). The chemokines CXCL9 and CXCL10 promote a protective immune response but do not contribute to cardiac inflammation following infection with *Trypanosoma cruzi*. Infect. Immun..

[36] Lee JH (2017). Pathogenic roles of CXCL10 signaling through CXCR3 and TLR4 in macrophages and T cells: Relevance for arthritis. Arthritis Res. Ther..

[37] Loos T (2006). TLR ligands and cytokines induce CXCR3 ligands in endothelial cells: Enhanced CXCL9 in autoimmune arthritis. Lab. Investig..

[38] Paula-Silva FW, Petean IB, da Silva LA, Faccioli LH (2016). Dual role of 5-lipoxygenase in osteoclastogenesis in bacterial-induced apical periodontitis. J. Endod..

[39] Kabashima H (2001). The presence of chemokine receptor (CCR5, CXCR3, CCR3)-positive cells and chemokine (MCP1, MIP-1alpha, MIP-1beta, IP-10)-positive cells in human periapical granulomas. Cytokine.

[40] Kwak HB (2005). Monokine induced by interferon-gamma is induced by receptor activator of nuclear factor kappa B ligand and is involved in osteoclast adhesion and migration. Blood.

[41] Yoneda N (2017). Development of a root canal treatment model in the rat. Sci. Rep..

[42] Sasaki H (2000). IL-10, but not IL-4, suppresses infection-stimulated bone resorption in vivo. J. Immunol..

[43] Fouad AF (1997). IL-1 alpha and TNF-alpha expression in early periapical lesions of normal and immunodeficient mice. J. Dent. Res..

[44] Venkataiah VS (2019). Periodontal regeneration by allogeneic transplantation of adipose tissue derived multi-lineage progenitor stem cells in vivo. Sci. Rep..

[45] Shima K (2018). C-X-C motif chemokine 12 enhances lipopolysaccharide-induced osteoclastogenesis and bone resorption in vivo. Calcif. Tissue Int..

[46] Takeshita S, Kaji K, Kudo A (2000). Identification and characterization of the new osteoclast progenitor with macrophage phenotypes being able to differentiate into mature osteoclasts. J. Bone Miner. Res..

[47] Garlet TP (2010). CCR2 deficiency results in increased osteolysis in experimental periapical lesions in mice. J. Endod..

[48] Rider D (2016). Elevated CD14 (cluster of differentiation 14) and toll-like receptor (TLR) 4 signaling deteriorate periapical inflammation in TLR2 deficient mice. Anat. Rec..

[49] Jenh CH (2012). A selective and potent CXCR3 antagonist SCH 546738 attenuates the development of autoimmune diseases and delays graft rejection. BMC Immunol..

[50] Metzemaekers M, Vanheule V, Janssens R, Struyf S, Proost P (2017). Overview of the mechanisms that may contribute to the non-redundant activities of interferon-inducible CXC chemokine receptor 3 ligands. Front. Immunol..

[51] Zhou J (2010). CXCR3-dependent accumulation and activation of perivascular macrophages is necessary for homeostatic arterial remodeling to hemodynamic stresses. J. Exp. Med..

[52] Oghumu S (2014). CXCR3 deficiency enhances tumor progression by promoting macrophage M2 polarization in a murine breast cancer model. Immunology.

[53] Kuan WP (2010). CXCL 9 and CXCL 10 as sensitive markers of disease activity in patients with rheumatoid arthritis. J. Rheumatol..

[54] Ji JD (2009). Inhibition of RANK expression and osteoclastogenesis by TLRs and IFN-gamma in human osteoclast precursors. J. Immunol..

[55] Hiyari S (2018). Genomewide association study identifies Cxcl family members as partial mediators of LPS-induced periodontitis. J. Bone Miner Res..

