# Supplementary material for: Inhibition of the CXCL9-CXCR3 axis suppresses the progression of experimental apical periodontitis by blocking macrophage migration and activation
Source: Sci Rep. 2021 Jan 28;11:2613. doi: 10.1038/s41598-021-82167-7 (PMC7844264; doi:10.1038/s41598-021-82167-7)
Supplement: Supplementary file 1 — Supplementary Information. [file 41598_2021_82167_MOESM1_ESM.docx]

**Inhibition of the CXCL9-CXCR3 axis suppresses the progression of experimental apical periodontitis by blocking macrophage migration and activation**

Tatsuya HASEGAWA^1^, V. VENKATA Suresh^1^, Yoshio YAHATA^1^, Masato NAKANO^1^, Shigeto SUZUKI^1^, Shigeki SUZUKI^2^, Satoru YAMADA^2^, Hideki KITAURA^3^, Itaru MIZOGUCHI^3^, Yuichiro NOIRI^4^, Keisuke HANDA^1,5^ and Masahiro SAITO^1^*

^1^Division of Operative Dentistry, Department of Ecological Dentistry, Graduate School of Dentistry, Tohoku University, Sendai, Miyagi, Japan.

^2^Division of Periodontology and Endodontology, Department of Ecological Dentistry, Graduate School of Dentistry, Tohoku University, Sendai, Miyagi, Japan.

^3^Division of Orthodontics and Dentofacial Orthopedics, Graduate School of Dentistry, Department of Community Social Dentistry, Tohoku University, Sendai, Miyagi, Japan.

^4^Division of Cariology, Operative Dentistry and Endodontics, Department of Oral Health Science, Graduate School of Medical and Dental Sciences, Niigata University, Niigata, Japan.

^5^Division of Oral Biochemistry, Department of Oral Science, Graduate School of Dentistry, Kanagawa Dental University, Yokosuka, Kanagawa, Japan

Corresponding Author:

Masahiro Saito, DDS, Ph.D., Professor

Tohoku University

Graduate School of Dentistry

Division of Operative Dentistry, Department of Restorative Dentistry

4-1 Seiryo-machi, Aoba-ku, 980-8575 Sendai, Miyagi, Japan

TEL: 81-22-717-8340

FAX: 81-22-717-8344

Email: mssaito@dent.tohoku.ac.jp

**Supplementary methods**

**Validation of tooth root development in the mouse mandibular 1st molar**

The left mandibular first molar was analyzed in 4 to 12 week-old mice that were sacrificed and scanned using a micro CT scanner (ScanX-mate-E90, Comscan Techno, Kanagawa, Japan) to measure the length and width of the mesial and distal roots. Three-dimensional (3D) image analysis software (TRI/3D-BON; Ratoc System Engineering, Tokyo, Japan) was used for quantitative analysis. The mesial root of the left mandibular first molar was used for this experiment.

**Quantification of the AP lesion volume by micro CT analysis**

The μCT voxel size was 5 μm. Quantification of the AP volume was determined by the range of the radiolucent area, based on CT data generated by TRI 3D-BON software. The apical area was determined at 0.5 mm from the bottom of the pulpal floor in the horizontal section, and the frontal section and sagittal section was extracted where the entire space fits into the alveolar bone. The apical radiolucency area was selected in frontal, sagittal and horizontal sections using the root tip as a reference. The AP lesion volumes were calculated from the radiolucent area of the subtraction image, based on the range of the radiopaque area determined by the software. Briefly, the window level and width were locked under the same scanning conditions. The periapical area was defined whereby the X-axis and Z-axis boundary was the region between the root surface and the alveolar bone surface, and the Y-axis boundary was the region between the alveolar bone surface and 0.5 mm apically from the pulp chamber. The lesion volume was expressed as cubic millimeters. Following micro CT analysis, the samples were processed for histological analysis.

**THP-1 cultures**

Human monocytic leukemia THP-1 cells were purchased from RIKEN Cell Bank (Tsukuba, Japan) and maintained in RPMI 1640 (Gibco; Thermo Fisher Scientific, Inc., Waltham, MA) supplemented with 5 mM L-glutamine, 100 IU/ml penicillin and streptomycin (FUJIFILM Wako Pure Chemical Corporation), and 10% FBS (Biowest, Nuaillé ,France), at 37°C and 5% CO_2_. Quantifications of migrated cells were performed using a VersaMax Microplate Reader in accordance with the manufacturer's protocol (Molecular Devices).

**Preparation of osteoclast precursors for osteoclast differentiation**

To isolate bone marrow cells, the femurs and tibiae were aseptically collected from 10 week-old male C57BL/6N mice. To obtain bone marrow cells, the epiphyses of these long bones were cut, and the bone marrow was flushed into a sterile Petri dish using a 25-gauge needle, and syringe prefilled with culture medium. The cell suspension was then filtered using a 40 μm nylon cell strainer (Corning Inc., Corning, NY) and centrifuged twice with minimal essential medium (α-MEM; Life Technologies). The harvested cells were then incubated in α-MEM containing 10% fetal bovine serum, antibiotics (100 IU/mL penicillin G and 100 μg/ml streptomycin; FUJIFILM Wako Pure Chemical Corporation), and macrophage colony stimulating factor (M-CSF). Non-adherent cells were removed by washing with PBS and adherent cells were harvested using a trypsin-EDTA solution (FUJIFILM Wako Pure Chemical Corporation). The harvested cells were seeded and further cultured in the presence of M-CSF until the cells reached confluence. These cells were then used as osteoclast precursors.

**Quantitative real-time RT-PCR analysis of inflammatory-related genes**

Total RNAs from cultured cells or a mandible with a periapical lesion were isolated using ISOGEN II (Nippon Gene, Tokyo, Japan) according to the manufacturer’s protocol. cDNA was synthesized from 1 µg of total RNA in a 20 µL reaction containing 10X reaction buffer, 1 mmol/L of a dinitrophenol phosphate (dNTP) mixture, 1 U/ L RNase inhibitor, 0.25 U/ L reverse transcriptase, and 0.125 mol/L random 9 meters (Takara, Tokyo, Japan). The cDNA was then amplified using specific mouse gene primers (sTable 1) and quantified in a real-time PCR apparatus (Bio-Rad CFX Connect, Applied Biosystems). The cycling conditions were as follows: 40 cycles at 95°C for 3 mins, 55°C for 30sec, 65°C for 5 sec. The expression of the tested genes was assayed using 2^-ΔΔCT^ method in comparison with the housekeeping gene GAPDH. The RT^2^ Profiler™ PCR Array (Qiagen, Frederick, MD) was used to screen a panel of 84 inflammatory cytokines and cytokine receptor genes (PAMM-011Z) or innate and adaptive immune response genes (PAMM-052Z) in the periapical area, and to determine the changes in gene expression after pulp exposure in the AP mouse model. Briefly, all samples were crushed using liquid nitrogen and total RNAs from the mandible jaw along and the periapical lesion were isolated at 3,7, 14 and 28 days after pulp exposure using ISOGEN II (Nippon Gene, Tokyo, Japan), used to synthesized cDNA with an RT^2^ First Strand Kit (Qiagen), and then amplified with 2X RT^2^ SYBR Green Mastermix (Qiagen) and CFX Connect (Bio-Rad Laboratories, Inc. Hercules, CA). The amplification protocol was as follows: 95°C for 10 min, then 40 cycles at 95°C for 15 sec and 60°C for 1 min. Clustergrams were generated using the RT^2^ PCR array data analysis web portal (https://dataanalysis2.qiagen.com/pcr) in accordance with the manufacturer’s instructions.

**Supplementary Fig. 1**


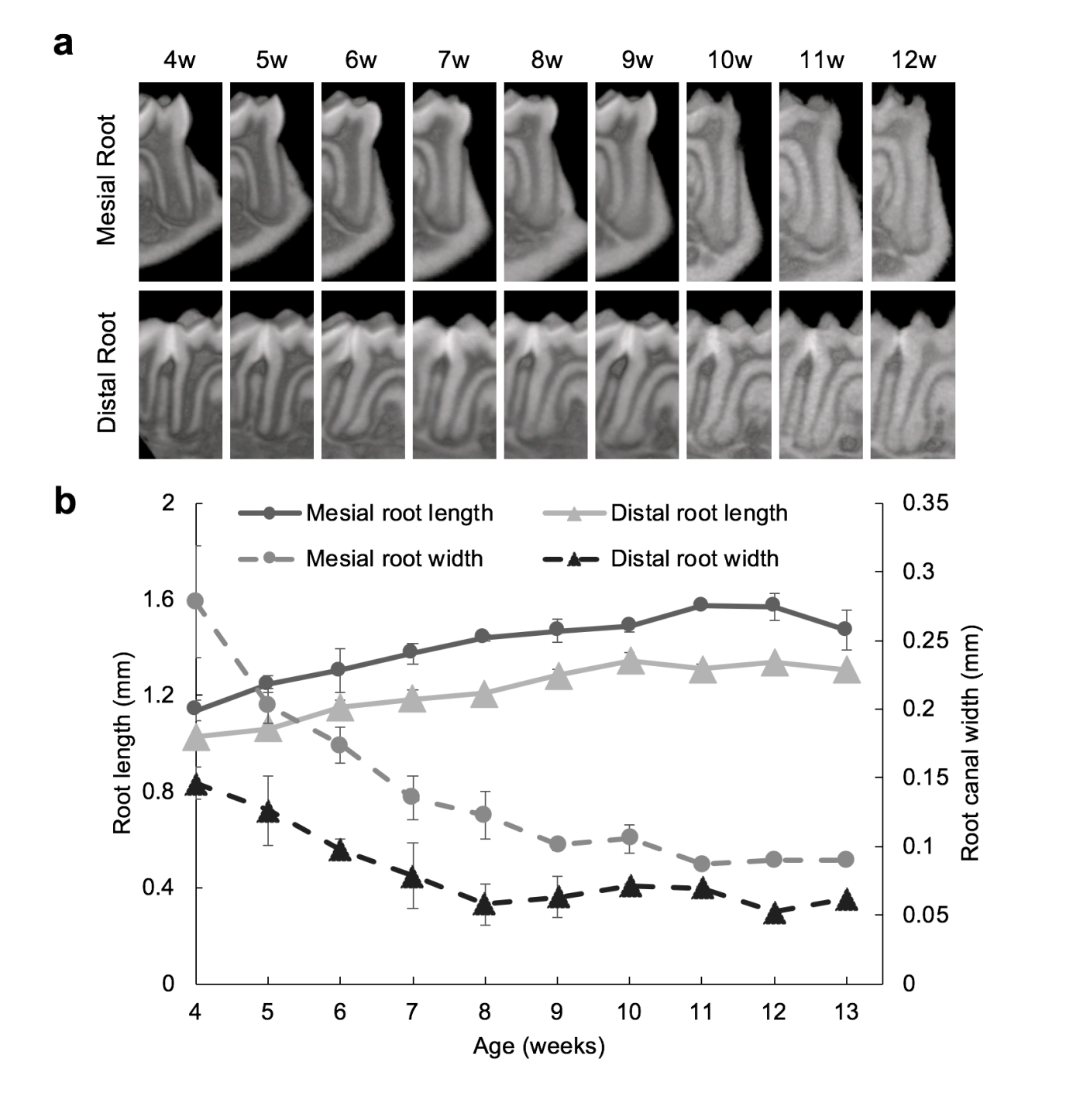


**Supplementary Fig. 2**


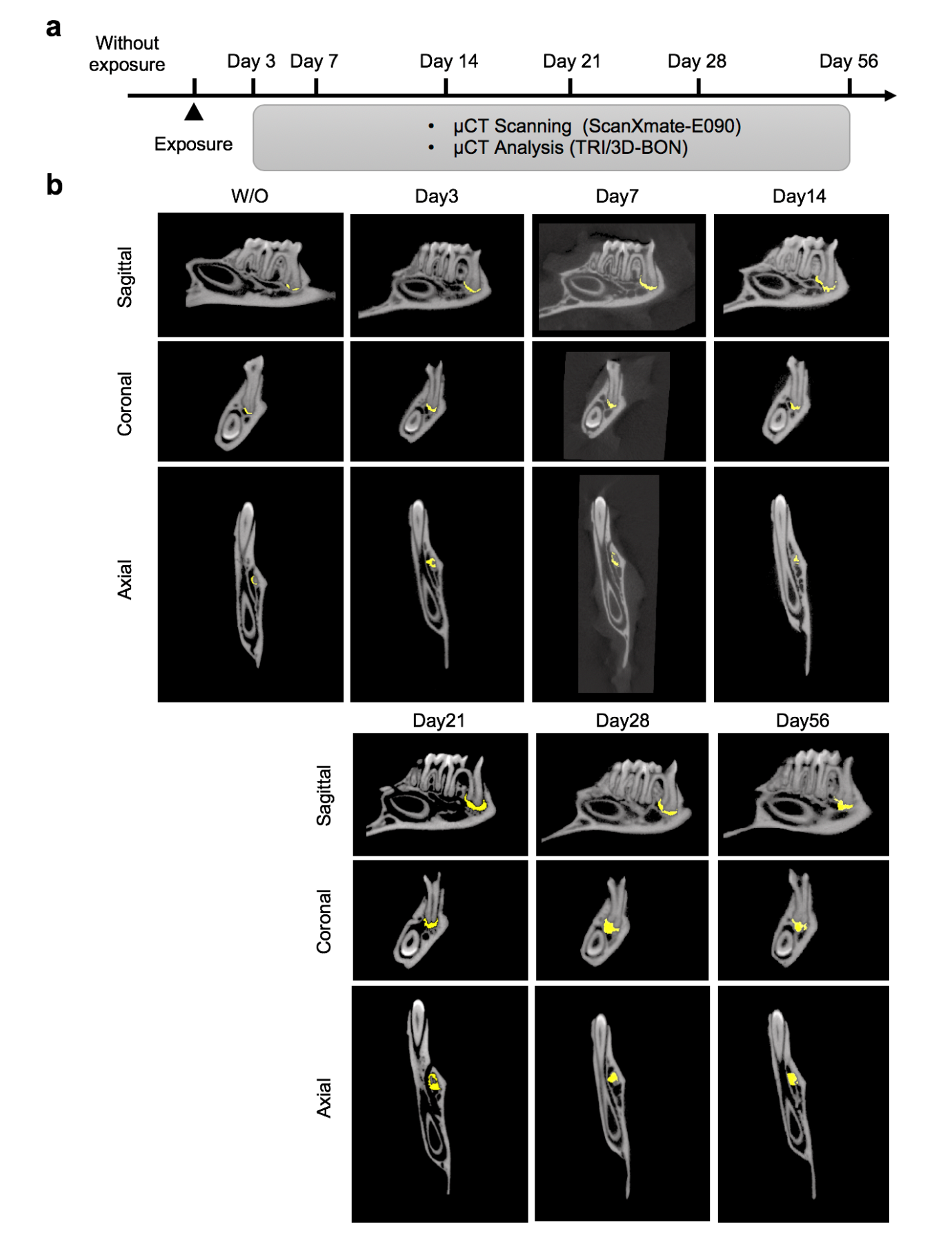


**Supplementary Fig. 3**


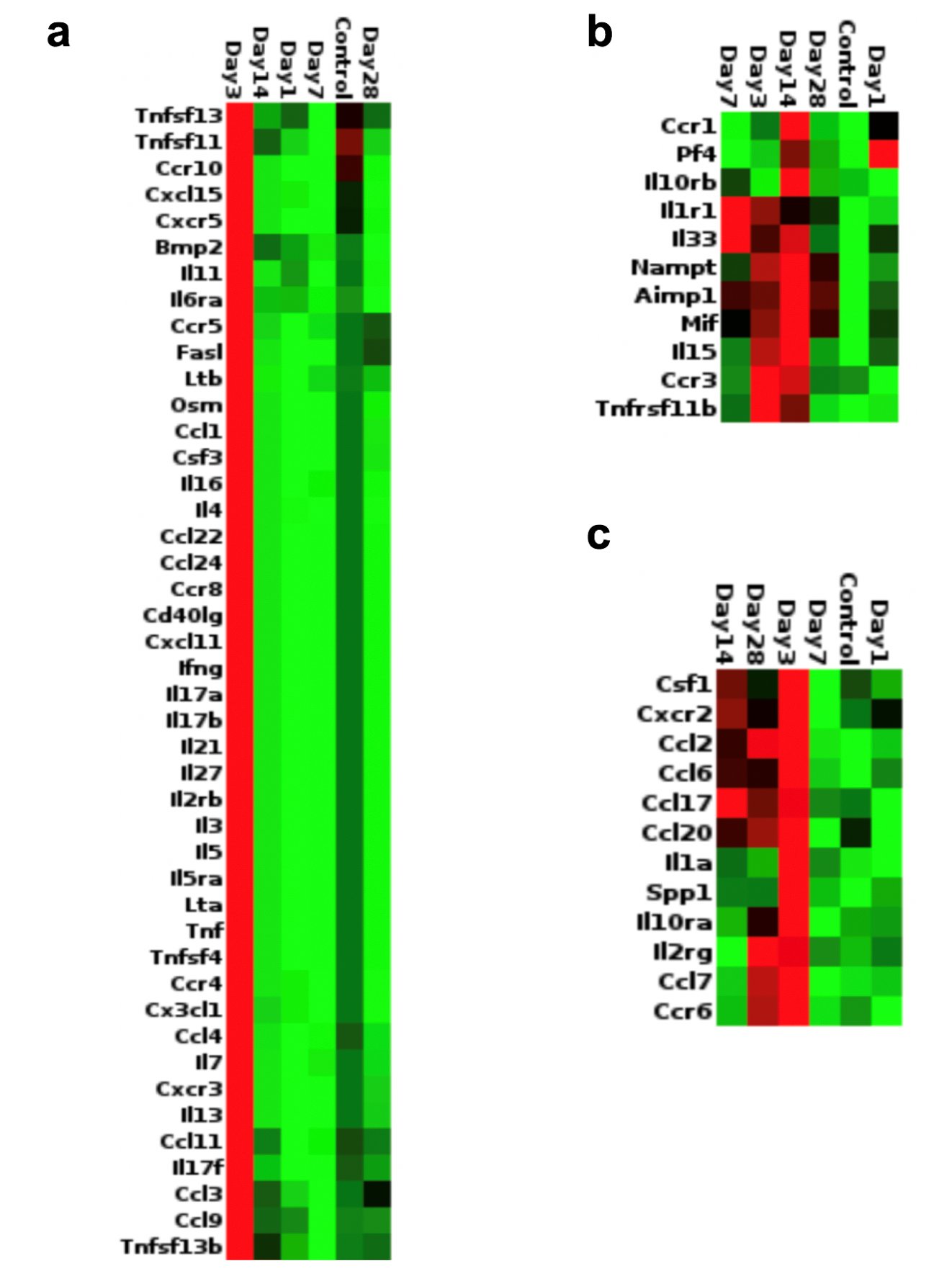


**Supplementary Fig. 4**


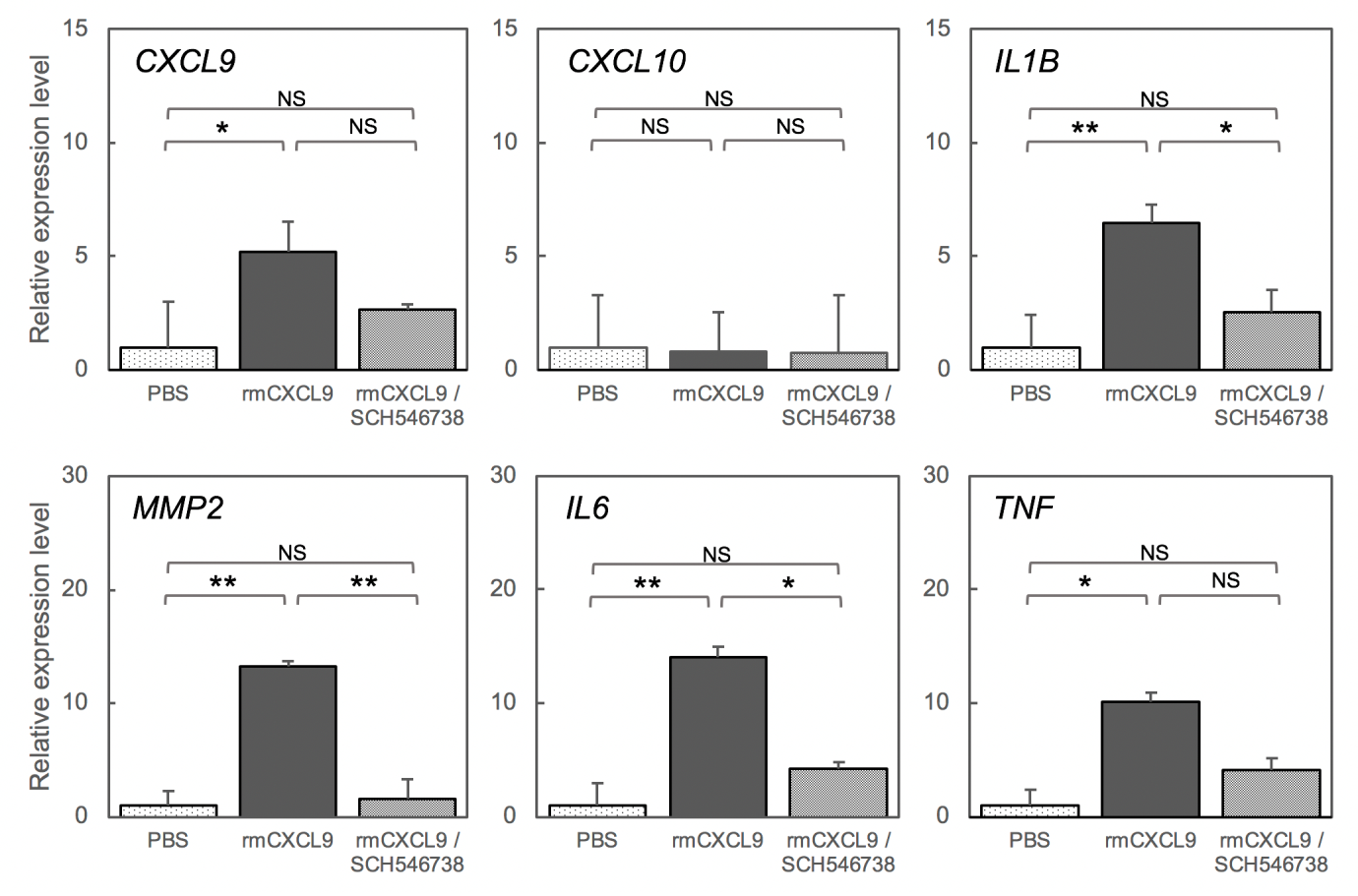


**Supplementary Fig. 5**


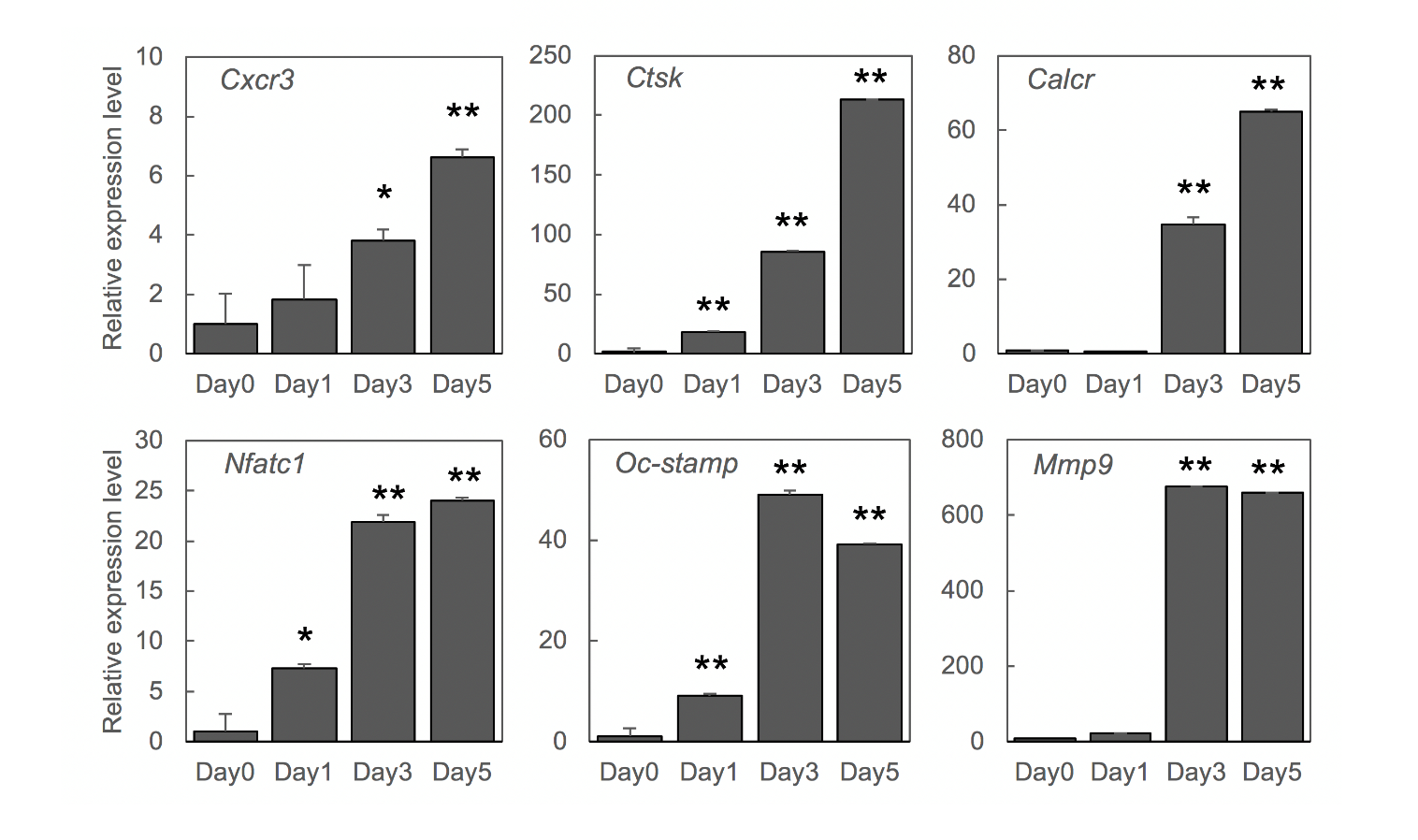


**Supplementary Fig. 6**


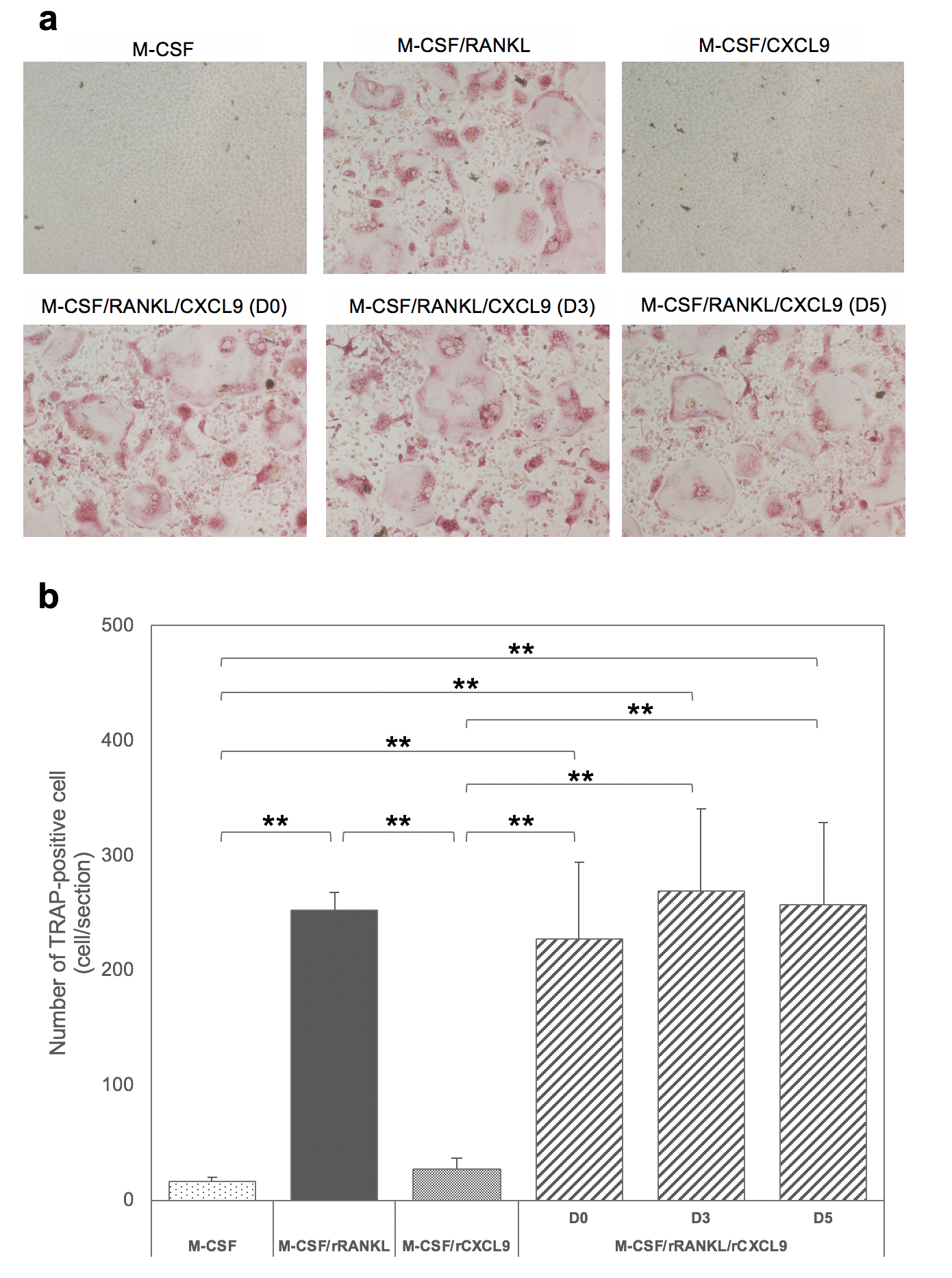


**Supplementary Figure Legends**

**Supplementary Figure 1**

**Validation of tooth root development in the mouse mandibular first molar.**

(a) Representative μCT images of the mesial and distal tooth root of the mouse mandibular first molar between 4 and 12 weeks. (b) Measurement of the root length (mm) and root canal width (mm) from μCT imagery as described in the methods section (mean ± SD, n=3 per group).

**Supplementary Figure 2**

**Quantification of AP lesion volumes by μCT analysis.**

(a) Experimental protocol for investigating AP development in a mouse model.
(b) Representative μCT images captured during the AP development of AP up to 56 days after pulp exposure. Radiolucent areas (sagittal, coronal and axial views) indicating a periapical lesion are highlighted in yellow and were detected using TRI/3D-BONE software based on the CT value.

**Supplementary Figure 3**

**Clustergram analysis of 84 inflammatory mediator and receptor genes expressed during AP development**

(a) Cluster of genes highly expressed at 3 days after pulp exposure. (b) Cluster of genes highly expressed between 1 to 14 days after pulp exposure. (c) Cluster of genes highly expressed at both 3 and 14 days after pulp exposure.

**Supplementary Figure 4**

**Suppression of the CXCL9-CXCR3 axis regulates the inflammatory response of THP-1 cells.** Validation of the inflammatory markers was conducted using real-time PCR analysis. Statistical analysis was conducted using a Turkey-Kramer test; *p<0.05, **p<0.01 (mean ± SD, n=4 per group).

**Supplementary Figure 5**

**Increased expression of CXCR3 during osteoclast differentiation.** The expression levels of CXCR3 and osteoclast markers including was analyzed in osteoclasts progenitors by quantitative PCR. Statistical comparisons were made with the Day0 group using the Dunnett test; *p<0.05, **p<0.01 (mean±SD, n=4 per group).

**Supplementary Figure 6**

**Effects of rmCXCL9 on osteoclast differentiation.** (a) Microscopic images of TRAP-positive cells. Osteoclast precursors were treated for 5 days with macrophage colony stimulating factor (M-CSF) alone, M-CSF/RANKL, M-CSF/CXCL9, or M-CSF/RANKL and rmCXCL9 together at 0, 3 and 5 days. (b) Numbers of TRAP-positive cells (cell/section). For quantification of TRAP positive cells, we selected 5 independent areas per one well and quantified using ImageJ software. Statistical analysis was conducted using he Turkey-Kramer test; *p<0.05, **p<0.01 (mean ± SD, 20 areas/n=4 per group).

**Supplementary Table 1**

Mouse and human gene primers used in the real time PCR analysis.

| 【Mouse】 | | |
| --- | --- | --- |
| *Gapdh* | F | CACTGAGCAAGAGAGGCCCTATCC |
|  | R | CCTAGGCCCCTCCTGTTATTATGG |
| *Nfkb1* | F | TCAGACACCTCTGCACTTGG |
|  | R | GCAGGCTATTGCTCATCACA |
| *Myd88* | F | GGCCTGAGCAACTAGGACTG |
|  | R | CGTGCCACTACCTGTAGCAA |
| *Tlr4* | F | GCTTTCACCTCTGCCTTCAC |
|  | R | GAAACTGCCATGTTTGAGCA |
| *Cd14* | F | GTCAGGAACTCTGGCTTTGC |
|  | R | GGCTTTTACCCACTGAACCA |
| *Tnf* | F | TATGGCTCAGGGTCCAACTC |
|  | R | CTCCCTTTGCAGAACTCAGG |
| *Il1b* | F | CAGGCAGGCAGTATCACTCA |
|  | R | AGCTCATATGGGTCCGACAG |
| *Il6* | F | AGTTGCCTTCTTGGGACTGA |
|  | R | CAGAATTGCCATTGCACAAC |
| *Cxcl9* | F | AAAATTTCATCACGCCCTTG |
|  | R | TCTCCAGCTTGGTGAGGTCT |
| *Cxcl10* | F | CCCACGTGTTGAGATCATTG |
|  | R | CACTGGGTAAAGGGGAGTGA |
| *Ccl5* | F | CCCTCACCATCATCCTCACT |
|  | R | CCTTCGAGTGACAAACACGA |
| *Ccl8* | F | GGGTGCTGAAAAGCTACGAG |
|  | R | TTCCAGCTTTGGCTGTCTCT |
| *Lgals3* | F | GATCACAATCATGGGCACAG |
|  | R | TGAATGGTTTGCCACTCTCA |
| *Mmp2* | F | TTTTTGTGCCCAAAGAAAGG |
|  | R | GCCCTCCTAAGCCAGTCTCT |
| *Ctsk* | F | CAGCTTCCCCAAGATGTGAT |
|  | R | AAGCACCAACGAGAGGAGAA |
| *Calcr* | F | CGGACTTTGACACAGCAGAA |
|  | R | CAGCAATCGACAAGGAGTGA |
| *Nfatc1* | F | TGGCCCCTATTCCTGTAGTG |
|  | R | CACTGAGCCAACTCCTCACA |
| *Oc-stamp* | F | CTGTGGTGCCAAACGTCTTA |
|  | R | TCTCCTGAGTGATCGTGTGC |
| *Mmp9* | F | TGAATCAGCTGGCTTTTGTG |
|  | R | ACCTTCCAGTAGGGGCAACT |

| 【Human】 |  |  |
| --- | --- | --- |
| *GAPDH* | F | AAGAGCACAAGAGGAAGAGAGAGAC |
|  | R | TTTATTGATGGTACATGACAAGGTG |
| *CXCL9* | F | TTTTCCTCTTGGGCATCATC |
|  | R | GAACAGCGACCCTTTCTCAC |
| *CXCL10* | F | CCAATTTTGTCCACGTGTTG |
|  | R | TTCTTGATGGCCTTCGATTC |
| *MMP2* | F | AGGGCACATCCTATGACAGC |
|  | R | ATTTGTTGCCCAGGAAAGTG |
| *MMP9* | F | TTGACAGCGACAAGAAGTGG |
|  | R | TCACGTCGTCCTTATGCAAG |
| *IL1B* | F | GCTGAGGAAGATGCTGGTTC |
|  | R | TCCATATCCTGTCCCTGGAG |
| *IL6* | F | AGGAGACTTGCCTGGTGAAA |
|  | R | CAGGGGTGGTTATTGCATCT |
| *TNF* | F | CCTGTGAGGAGGACGAACAT |
|  | R | AGGCCCCAGTTTGAATTCTT |
